# In Vivo Application of Carboranes for Boron Neutron Capture Therapy (BNCT): Structure, Formulation and Analytical Methods for Detection

**DOI:** 10.3390/cancers15204944

**Published:** 2023-10-11

**Authors:** Tainah Dorina Marforio, Andrea Carboni, Matteo Calvaresi

**Affiliations:** Dipartimento di Chimica “Giacomo Ciamician”, Alma Mater Studiorum—Università di Bologna, Via Francesco Selmi 2, 40126 Bologna, Italy; tainah.marforio2@unibo.it

**Keywords:** carboranes, metallacarboranes, COSAN, boron neutron capture therapy (BNCT), boron agents, chemical conjugates, delivery systems, in vivo, cancer, anticancer treatments

## Abstract

**Simple Summary:**

Boron neutron capture therapy (BNCT) is an emerging anticancer treatment. The success of BNCT relies on the delivery of adequate quantities of ^10^B to tumor cells. Carboranes, polyhedral clusters containing boron, carbon and hydrogen atoms, are promising candidates as boron agents in BNCT. In this review, we provide an overview and state-of-the-art description of applications of carboranes for in vivo BNCT studies. We comprehensively report: (i) the different molecules used, (ii) their formulation and administration strategies, (iii) the tumor models investigated and (iv) qualitative and quantitative methodologies for carborane detection in in vivo experiments.

**Abstract:**

Carboranes have emerged as one of the most promising boron agents in boron neutron capture therapy (BNCT). In this context, in vivo studies are particularly relevant, since they provide qualitative and quantitative information about the biodistribution of these molecules, which is of the utmost importance to determine the efficacy of BNCT, defining their localization and (bio)accumulation, as well as their pharmacokinetics and pharmacodynamics. First, we gathered a detailed list of the carboranes used for in vivo studies, considering the synthesis of carborane derivatives or the use of delivery system such as liposomes, micelles and nanoparticles. Then, the formulation employed and the cancer model used in each of these studies were identified. Finally, we examined the analytical aspects concerning carborane detection, identifying the main methodologies applied in the literature for ex vivo and in vivo analysis. The present work aims to identify the current strengths and weakness of the use of carboranes in BNCT, establishing the bottlenecks and the best strategies for future applications.

## 1. Introduction

**Figure 1:**
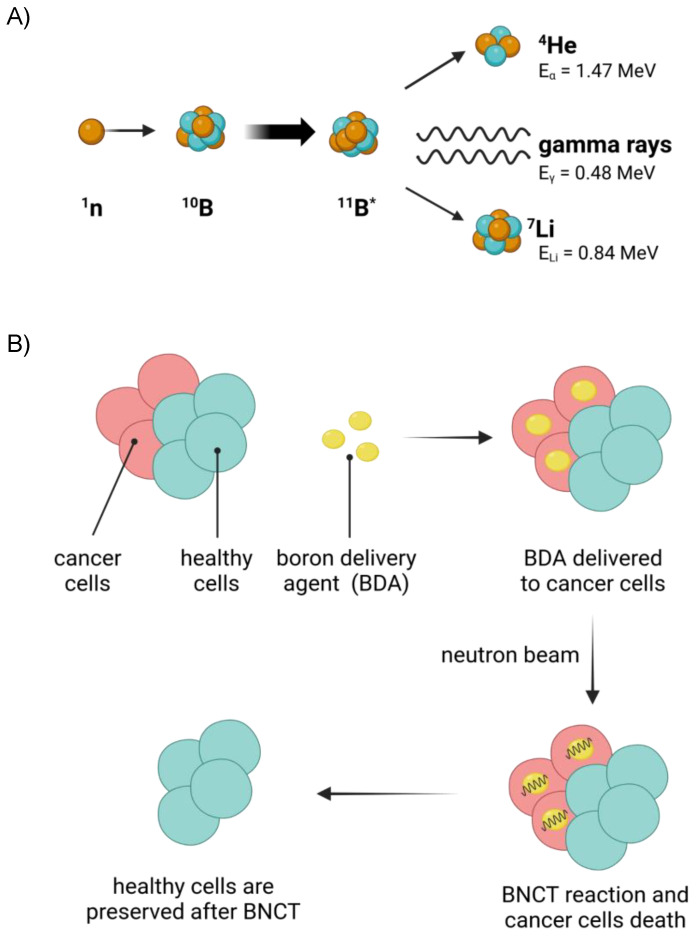
Schematic representation of (**A**) nuclear fission reaction on ^10^B atom and (**B**) BNCT mode of action. Images created with BioRender.com.

Boron neutron capture therapy (BNCT), first proposed by Gordon Locher in 1936, is a binary radiotherapeutic methodology for cancer treatment. The technique relies on the nuclear fission reaction triggered by low-energy thermal neutrons that hit boron atoms previously accumulated in cancerous cells. ^10^B stable isotopes irradiated with thermal (0.025 eV) or epithermal (0.5 eV to 40 keV) neutrons beam results in ^10^B(n, α)^7^Li nuclear reactions, generating α-particles (^4^He, of ~1.47 MeV) and recoiling ^7^Li nuclei (~0.84 MeV) (Figure 1). These particles possess high linear energy transfer (LET), in the order of ~175 keV μm^−1^, and the excited recoiling ^7^Li nuclei generally emit a low LET γ-ray during deexcitation. The generated Li- and α-particles are characterized by high energy and short travel distances of <5 μm and <10 μm, respectively. Hence, in biological systems, the process can result in extremely localized lethal effects in areas in the range of a human cell (~10 μm diameter).

Consequently, BNCT is a highly (cell) selective approach in cancer therapy, able to destroy only those cells that have accumulated enough ^10^B, sparing the surrounding healthy tissues. Such an enhanced selectivity can result in less severe side-effects compared to other anticancer therapies, promoting BNCT as a precision medical approach for the treatment of several types of tumors [1,2,3]. BNCT is indicated for head and neck tumors and brain tumors that are difficult to remove surgically and cutaneous melanomas [4,5,6,7,8,9,10]. To date, BNCT has been tested in phase I/II trials in several countries, such as the USA, Finland, the Czech Republic, Sweden, Japan, Taiwan, Argentina, Germany, Italy, Poland and China [11]. In May 2020, BNCT was authorized by the Japanese National Health Insurance system for the treatment of recurrent head and neck cancers [12].

The development of accelerator-based neutron sources with effective beam intensity for BNCT allows the installation of BNCT facilities in situ, overcoming one of the major obstacles for the bench-to-bedside transition of the BNCT, i.e., access to a nuclear reactor in the hospital where patients would undergo the treatment [13,14,15]. Alternative radiation sources, such as X-rays, have also been employed in attempts of generating novel therapeutic strategies for boron-based radiation therapy [16].

A remaining issue is the selective delivery of boron to cancer cells. In particular, boron agents should meet the following requirements: (i) approximately 10^9 10^B atoms should be delivered per cancer cell (~20–40 μg [B]/g[tumor]) [17], (ii) this concentration should be retained for several hours and stay constant during the thermal neutron irradiation, (iii) high tumor/blood (T/B > 3.5) and tumor/normal tissue (T/N ≥ 3) boron concentration ratios should be obtained [17] and (iv) there should be a low toxicity and fast clearance from healthy tissues and blood after the treatment. In addition, the accumulation of boron inside the tumor is not enough. To achieve effectiveness of BNCT, boron must be delivered inside the cells, only with intracellular localization; the DNA of the cell and organelles are damaged upon irradiation.

In current BNCT clinical trials, boron is generally delivered to the tumors using boronphenylalanine (BPA or Borofalan or Steborinine^®^) and sodium borocaptate (BSH) [18].

The maximum value of boron concentration in tumors obtained in clinical studies was 36.9 µg/g for BPA [19] and 19.9 µg/g for BSH [20].

BPA is extremely effective because its structure mimics the characteristics of endogenous phenylalanine and it is selectively uptaken by cancer cells because it is specifically recognized by L-type amino acid receptors (i.e., LAT1) that are generally overexpressed in cancer cells [21,22]. The synthesis of enantiomerically pure BPA can be obtained in excellent yield, under mild conditions [23,24,25,26]. However, some drawbacks exist, such as: (i) the presence of free hydroxyl groups can lead to the formation of reversible covalent bonds with endogenous carbohydrate molecules through boronate ester formation, (ii) low solubility (1.6 g L^−1^), (iii) metabolic instability of the boronic acid when exposed to reactive oxygen species (ROS) naturally present in the cells and especially in cancer cells and (iv) accumulation in healthy cells because BPA is a derivative of a natural amino acid and can take part in protein synthesis [18].

BSH increases the number of boron atoms in a single compound, and it is characterized by a high chemical and catabolic stability [18]. On the other side, (i) BSH can undergo dimerization, producing BSSB, (ii) it lacks receptor-mediated selective transportation for cancer treatments, (iii) the pharmacokinetics and bioavailability of BSH is poor and (iv) its synthesis is not cost-effective.

A great effort has been dedicated to the development and in vivo testing of BPA derivatives, such as BPA-F, a conjugate of BPA with fructose [27,28,29,30,31,32,33,34] and closo-dodecaborate derivatives [35,36,37,38,39,40,41,42,43,44,45,46,47,48,49,50] readily available via alkylation of BSH or via the nucleophilic opening of the cyclic oxonium derivatives to bypass their inherent limitations [18].

Carboranes are a family of boron-rich chemicals, which makes them promising candidates for the role of boron agents in BNCT [51,52,53,54,55]. They consist of polyhedral clusters containing boron, carbon and hydrogen atoms arranged in a variety of scaffolds differing in structure and composition. If the polyhedra is complete, the term closo- is used; if one, two or three vertexes are missing, nido-, arachno- and hypho- terms are used [56].

Dicarba-closo-dodecaboranes (C_2_B_10_H_12_), simply referred to as closo-carboranes, are the most stable and applied members of the family. Similar to benzene nomenclature, three isomers are identified depending on the relative position of the two carbon atoms: ortho-(1,2-C_2_B_10_H_12_), meta-(1,7-C_2_B_10_H_12_) and para- (1,12-C_2_B_10_H_12_) carborane. The meta- and para-carboranes can be obtained by thermal degradation of the ortho- isomer [56]. The 3D-aromaticiy of the resulting cages confers a high stability to these chemicals [57,58].

**Figure 2:**
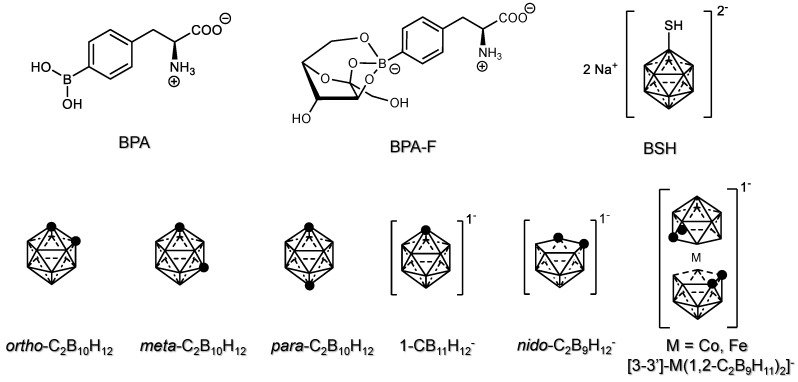
Structures of borophenylalanine (BPA), BPA−fructose complex, mercaptoundecahydrododecaborate (BSH), closo-carborane (ortho-, meta-, and para-C_2_B_10_H_12_), closo-dodecaborate anion (1-CB_11_H_12_^−^), nido-carbonane anion (nido-C_2_B_9_H_12_^−^), COSAN ([3-3′]-Co(1,2-C_2_B_9_H_11_)_2_]^−^) and FESAN ([3-3′]-Fe(1,2-C_2_B_9_H_11_)_2_]^−^).

The open-cage nido-carborane [7,8-C_2_B_9_H_12_]^−^ is typically obtained by deboronation of ortho-carborane, e.g., after reaction with a strong Lewis base such as alkoxides, amines, fluorides and recently N-heterocyclic carbene [59]. As shown in Figure 2, the further deprotonation to nido-carborane [7,8-C_2_B_9_H_11_]^2−^ and treatment with CoCl_2_ leads to the synthesis of cobalt (III) bis(dicarbollide) anion [56]. This sandwich-like metallacarborane, referred to as COSAN, possesses a C2h symmetry. Varying the metal center, other metalla-carborane can be also synthesized [56].

In addition to the high number of boron atoms, all these carborane structures possess interesting pharmacophoric features, i.e., they are hydrophobic, aromatic, hydrogen bond acceptors/donors and due to their abiotic nature (i.e., synthetic chemicals) they are extremely stable in living organisms, which makes them interesting for medical applications [60,61,62] and in particular for BNCT [59,63,64,65,66].

Furthermore, carboranes possess acidic C-H bonds and hydridic B-H groups that can be functionalized in order to attach novel moieties to their structures [56,67,68,69,70,71]. Such functionalizations are usually aimed at decreasing carboranes’ lipophilic character, which currently hampers their clinical translation.

In the last decades, extensive efforts in the scientific community have led to the synthesis of hundreds of novel carborane-based derivatives and their testing as boron agents for BNCT. Many in vivo studies have demonstrated the potential transition of carboranes from laboratory to clinical trials. Such in vivo studies allowed the testing of carboranes in BNCT but also the assessment of several endpoints, including their fate (i.e., stability, partitioning, pharmacokinetic) and effects (i.e., bioaccumulation, toxicity).

The present review focuses on such in vivo investigations with the aim of providing a comprehensive overview of the published literature and summarizing (i) the strategies for carborane derivatization or incorporation in delivery systems, (ii) their formulation and administration modality and (iii) the analytical strategies for their quantification in vivo.

## 2. In Vivo Studies of Carboranes for BNCT

The present section focuses on the structure, formulation, administration and cancer models used in in vivo studies employing carborane/carborane derivatives.

The hydrophobicity of pristine carboranes hinders their direct in vivo administration. To overcome this limitation, two main approaches have been used: (i) the synthesis of water-soluble carborane derivatives by the chemical functionalization of their structure and (ii) the use of delivery systems able to mediate their transport in biological media.

### 2.1. Chemical Derivatization of Carboranes for In Vivo Studies

Carboranes are completely insoluble in water. Installing hydrophilic moieties on the carborane cages (i) improves their solubility in water, (ii) defines novel pharmacokinetic, pharmacodynamic and physiochemical profiles and (iii) facilitates their administration in living organisms. In addition, the derivatization of carboranes with specific moieties (i.e., targeting or imaging agents) also enhances their cellular uptake and selectivity against cancer cells and enables the use of specific analytical techniques for their detection. A list of the studies employing such strategies, with regard to the derivative category and the specific molecules applied, is provided in the Supplementary Materials (Table S1).

Different functionalization strategies were developed to target the C-H and B-H bonds present on the carborane. The presence of acidic C-H groups allows its deprotonation by a strong base, obtaining a nucleophilic vertex that can perform a nucleophilic attack on the electrophile molecule of interest. The most common strategy foresees the use of n-BuLi (n-butyllithium) [56,67]. However, n-BuLi is an extreme reactive compound and it is not compatible with many functional groups; therefore, although hundreds of carborane derivatives have been synthesized in the last few decades using this synthetic strategy, their structures are quite similar from the chemical point of view. Alternative synthetic approaches, such as the use of carbyne intermediates [68], allowed the synthesis of more complex structures.

In general, the functionalization of the carboranes at the boron vertexes is much more challenging than C-H because it is less site-selective and B–H bonds are much less reactive than C–H. However, the intensive work in carborane derivatization, mainly based on (transition) metal catalysis [69,70,71,72], i.e., the metal-catalyzed activation of the B-H bond or cross-coupling at the B-X (X = Br or I) bond, is making carborane functionalization more accessible and easier, advancing the complexity of structures containing carboranes and in that way advancing BNCT.

#### 2.1.1. Carbohydrate Derivatives of Carboranes

Carbohydrates are polyhydroxylated compounds and their conjugation to carboranes highly enhances their solubility, leading to an easier dissolution in body fluids and increased bioavailability, ultimately leading to better therapeutic outcomes. Carbohydrates are able to target specific tissues by the recognition of certain receptors present on the surface of cells. Therefore, attaching carbohydrates to carboranes can enhance the selectively towards these tissues, increasing the concentration of the drug in the desired site [73,74,75]. Being involved in various immune recognition processes, the attachment of carbohydrates can also modulate the immune response, potentially reducing adverse effects and increasing the efficacy of the drug [76,77,78].

**Figure 3:**
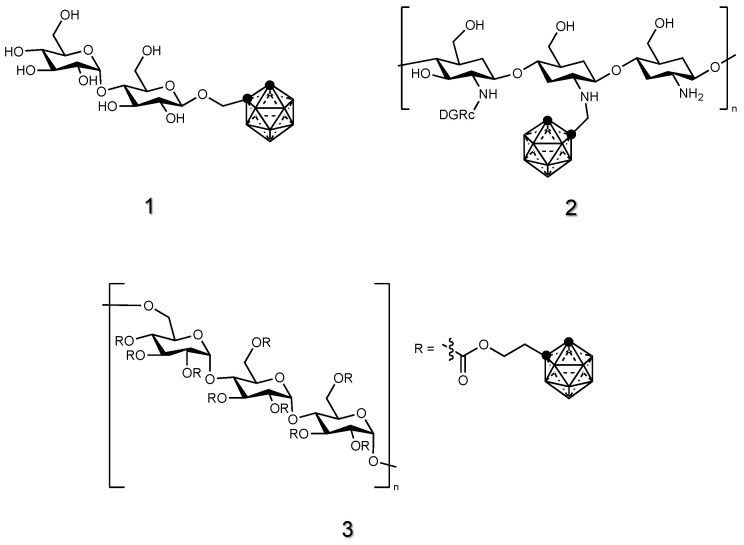
Structures of carboranyl-maltoside (**1**), cRDG-COS-carborane (**2**) and carborane-bearing pullulan (**3**).

Despite the great effort reported in the literature regarding the synthesis and in vitro testing of carbohydrate derivatives of carboranes [73,79,80,81,82], the examples of such compounds tested in vivo are few [83,84,85,86]. Ortho-carboranyl glycosides of glucose, maltose and lactose were synthetized and tested in vitro for their toxicity toward C-6 rat glioma cells after thermal neutron treatment [87], but only the carboranyl maltoside compound was tested in vivo [88]. This carborane derivative **1** (Figure 3) displayed a boron uptake in murine induced tumors higher than BSH. Further in vivo tests involved a carborane derivatized with a chitosan oligosaccharide (COS) [84] **2** (Figure 3), which promoted the formation of nanoparticles, leading to carborane dispersion and inducing their phagocytosis by cells. Pullulan, a linear polysaccharide consisting of repeating units of maltotriose, has been covalently linked to ortho-carborane **3** (Figure 3) to obtain a safe and effective platform to deliver boron to fibrosarcoma through the formation of nanogels [85,86].

#### 2.1.2. Nucleoside Derivatives of Carboranes

**Figure 4:**
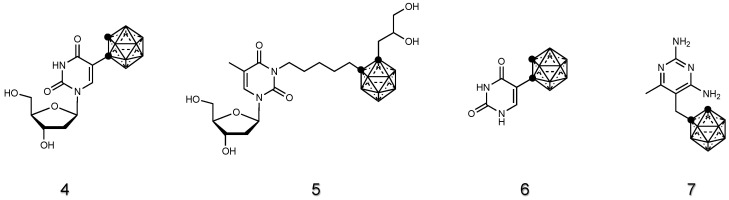
Structures of deoxyuridine- (**4**), thymidine- (**5**), uracil- (**6**) and pyrimidine-carborane (**7**) derivatives.

Nucleosides can be recognized and taken up by cells through nucleoside transporters present on the membrane. Thus, designing and synthesizing boron-containing nucleosides is justified for their potential to specifically accumulate in rapidly dividing tumor cells. By attaching these moieties to carboranes, the resulting conjugate can exploit the cellular uptake pathways already in place for nucleosides, facilitating the entry of carborane into cells. This can be particularly advantageous when targeting specific sites, such as cancer cells. For these reasons, various types of carborane-nucleosides derivatives [89], such as deoxyuridine [90,91,92,93], thymidine [94,95,96,97], uracil [98] and analogues [99], were synthesized and their potential in BNCT was assessed in vivo (**4**–**7**, Figure 4).

Special attention was given to thymidine derivatives due to their ability to target the enzyme thymidine kinase 1 (TK1), which is overexpressed in different cancers. The 3-[5-{2-(2,3-Dihydroxyprop-1-yl)-o-carboran-1-yl}pentan-1-yl]thymidine (N5-2OH) [94,95,96,97] and 5-ortho-carboranyl-2′-deoxyudirine [90,91,92,93] compounds have been widely tested on rat glioma and emerged as valid candidates for BNCT. However, a drawback of such nucleotide-carborane derivatives is that their administration usually requires a variable amount of organic solvent to allow the complete solubilization.

#### 2.1.3. Drug Derivatives of Carboranes

Another strategy to enhance the solubility and the targeting ability of carboranes is the conjugation with drugs that are known to possess anticancer or targeting properties. The novel dual compounds displayed unique biological and physicochemical properties that complement the pharmacological and targeting activities of both the drugs and carboranes.

**Figure 5:**
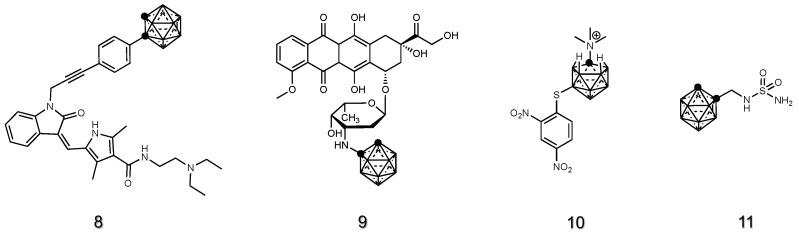
Structures of sunitinib- (**8**), doxorubicin- (**9**), nitroimidazole- (**10**) and sulfonamide-carborane (**11**) derivatives.

Tyrosine kinase inhibitors (TKIs) specifically target and inhibit the activity of tyrosine kinases, which are enzymes highly expressed on cancer cells and implicated in the process of cancer formation and growth. TKIs are widely used to treat cancers due to their role in carcinogenesis [100]. Sunitinib and erlotinib, well-established TKIs [101,102], work by targeting and inhibiting multiple tyrosine kinases receptors, including vascular endothelial growth factor receptors (VEGFRs), platelet-derived growth factor receptors (PDGFRs) and stem cell factor receptors (KIT). By blocking these kinases, sunitinib and erlotinib interfere with signaling pathways involved in tumor growth, angiogenesis (formation of new blood vessels) and metastasis (spread of cancer). Sunitinib [101] and erlotinib [103] have been conjugated to ortho- and meta-carboranes, as well as to COSAN, in order to develop bifunctional compounds that showed tyrosine kinase inhibition and boron accumulation in cancer cells for BNCT application. Among them, sunitinib-meta-carborane hybrid **8** (Figure 5) was tested in vivo for anti-glioblastoma activity in immunosuppressed mice bearing human U87 MG tumors.

Doxorubicin (DOX) displays the interesting features of being a potent chemotherapy drug, widely used in the treatment of various types of cancers, but also a chromophore, able to absorb light in a wide range of the visible spectrum [104]. The nuclear translocation property of DOX was exploited by covalently attaching doxorubicin to carborane **9** (Figure 5) in order to concentrate boron in glioma tumor-bearing mice [105].

In solid tumors, unique microenvironments can be found, often presenting regions of hypoxia due to inadequate oxygen supply. Reductive cytotoxic agents, such as nitroimidazoles, exhibit preferential accumulation in such hypoxic areas of tumors due to the presence of a nitro group (-NO_2_) that can undergo reductive activation under conditions of low oxygen tension [106]. In well-oxygenated tissues, nitroimidazoles are relatively stable and have low reactivity. However, within hypoxic regions of tumors, the lack of oxygen hinders normal cellular metabolic processes, leading to the presence of reducing agents, such as nitroreductase enzymes. These enzymes reduce the nitro group of nitroimidazoles, resulting in the formation of highly reactive intermediates that immediately react with biomolecules such as DNA, proteins and lipids. One of the earliest reports found in the literature about the in vivo testing of nitro-containing carborane is the work of Morris et al., where 7-(CH_3_)_3_N-nido-carborane was derivatized with a nitroaromatic system [107], **10** (Figure 5). This molecule was efficiently incorporated into melanoma and its clearance from blood was reported to be adequate for BNCT requirements. In a later study, a nitroimidazole-carborane linked to a polyether-isoxazole (or a polyether-carbamate) was also tested in squamous cell carcinoma or sarcoma-bearing mice [108].

Within solid tumors’ hypoxic regions, acidic microenvironments can also be developed. Such conditions can lead to the overexpression of carbonic anhydrase IX (CAIX), an important enzyme that regulates the acid-base balance and pH. Due to its unique expression pattern and involvement in cancer progression, CAIX has emerged as a potential target for therapeutic intervention in certain types of tumors [109]. Sulfonamides are a class of CAIX inhibitors whose structure resembles that of carbonic acid (H_2_CO_3_), the natural substrate of CAIX. In 2020, a sulfonamido-functionalized carborane **11** (Figure 5) was tested as a dual compound able to simultaneously inhibit CAIX and deliver boron to mesothelioma and breast cancer [110].

#### 2.1.4. Porphyrin Derivatives of Carboranes

Porphyrins are a family of conjugated macrocyclic compounds, based on four pyrrole units linked together, displaying a high potential for cancer treatment due to their unique properties [111,112]. These macrocycles tend to selectively accumulate in cancer cells. In addition, given their fluorescent properties, porphyrin derivatives can also be used as imaging agents for fluorescence imaging techniques.

Accordingly, the functionalization of carboranes with porphyrins showed potential benefits, such as (i) targeted delivery to tumor cells, minimizing the exposure of healthy tissues, (ii) increased cellular uptake of the dual drug, (iii) controlled release, since porphyrins can be designed to be stimuli-responsive to light, pH or enzymes, and (iv) theranostic applications exploiting the imaging properties of porphyrin.

**Figure 6:**
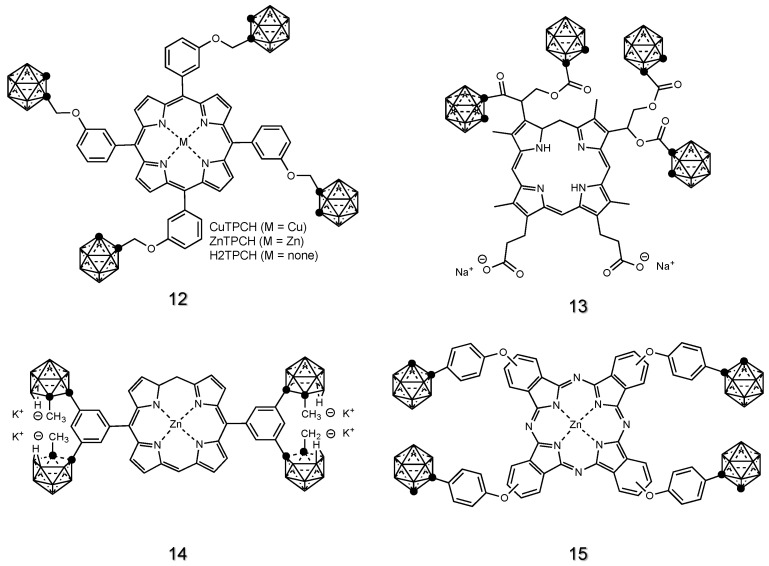
Structures of CuTCPH, ZnTCPH and H2TCPH (**12**), BOPP (**13**), ZnB_4_Pc phtalocyanin (**14**) and TPFC (**15**), containing carborane moieities.

From the beginning of 1990, Miura and colleagues explored the potential of lipophilic porphyrins containing boron for in vivo applications [43,113,114,115,116,117,118,119,120,121,122,123,124,125,126,127]. Since then, significant progress has been made in the development of a series of porphyrin-based drugs [128,129], also complexed with metals such as Zn and Cu. The incorporation of these metal atoms into porphyrins can influence their biological activity and allow the use of radionuclides (i.e., ^64^Cu) for tracing and quantifying the boron distribution during in vivo treatment. The functionalization of the porphyrin scaffold led to the synthesis of several compounds tested for in vivo applications in BNCT, including CuTCPH [116,117,118,119,122,124,126,130], ZnTCPH [122], H2TCP [131,132,133,134,135] and BOPP [136,137], whose structures (**12-13**) are reported in Figure 6.

Phtalocyanine derivatives were also synthesized and tested in vivo: the first example dates to 2005, when Friso et al. reported the use of tetra-carboranyl-methylphenoxy-substituted Zn(II)-phtalocyanine **14** (ZnB_4_Pc) on melanotic melanoma (Figure 6) [138]. Other studies focused on the synthesis of carboranyl-containing chlorin **15** (TPFC), (Figure 6), to develop a dual sensitizer for BNCT and photodynamic therapy (PDT) for the treatment of malignant glioma [125,139]. Here, the high fluorescence of the chlorine core bound to carborane allowed the authors to perform a fluorescence-guided detection and resection of the tumor in mice.

#### 2.1.5. Imaging Agent Derivatives of Carboranes

**Figure 7:**
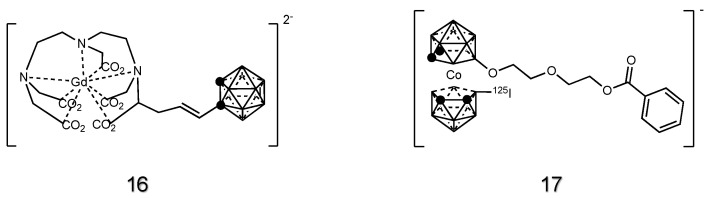
Structures of magnevist-carborane (**16**) and COSAN-iodinated (**17**) derivatives.

Pristine structures cannot be detected by several commonly applied imaging techniques. To perform real-time visualization or quantification of the carborane uptake and selectivity in tumors, several studies functionalized the cage by covalently binding imaging agents, such as fluorescent dyes, contrast agents or radiopharmaceuticals. These include the porphyrin derivatives discussed above. Other derivatizations were achieved with gadolinium-chelates [108,140,141,142] that are commonly used as contrast agents in magnetic resonance imaging (MRI), **16** (Figure 7), allowing the real-time localization of the carborane derivatives during in vivo experiments.

Iodine radioisotopes [143,144,145,146,147] **17** (Figure 7) were also conjugated to the carboranes and used as radiotracers for boron quantification and localization during in vivo BNCT experiments.

#### 2.1.6. Amino Acid Derivatives of Carboranes

**Figure 8:**
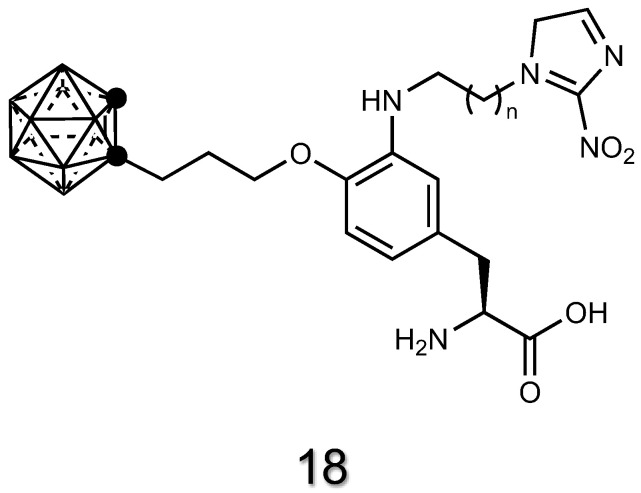
Structures of phenylalanine-carborane derivative (**18**).

Specific membrane transport proteins exist that carry amino acids across cell membranes [148]. For instance, the protein transporter Large-neutral Amino Acid Transporter 1 (LAT-1) is responsible for conveying amino acids such as phenylalanine and tyrosine. LAT-1 is overexpressed in many different tumor types and can be addressed to develop cancer-targeted delivery systems [21]. The ortho-carborane derivative of phenylalanine **18** (Figure 8) exploits the selectivity of phenylalanine moiety toward LAT-1 [149].

#### 2.1.7. Peptide Derivatives of Carboranes

Peptides are widely employed for targeted drug delivery, since they can be designed to specifically recognize and bind to certain receptors present on the surface of cells, including cancer cells. In addition, peptides are generally more biocompatible and less immunogenic compared to other targeting agents. For these reasons, different peptide sequences were incorporated into the boron delivery system for BNCT [150], and some peptide-carborane derivatives were synthesized [147,151,152].

**Figure 9:**
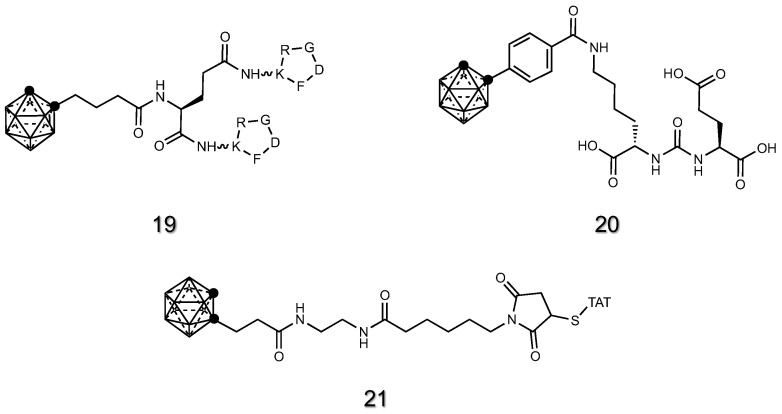
Structures of cRGD- (**19**), PSMA- (**20**) and TAT (GRKKRRQRRRPQ)- (**21**) carborane derivatives.

Cyclic arginine-glycine-aspartate (cRGD) is the most common aminoacidic sequence able to bind integrins on the extracellular matrix (ECM). Integrins are overexpressed in many cancer cells, and carborane derivatives conjugated to a cRGD peptide **19** (Figure 9) showed a selective antitumor activity and enhanced accumulation in cancer cells during in vivo experiments [46].

Prostate-specific membrane antigen (PSMA) is significantly expressed in prostate cancer cells. In the last years, numerous PSMA-targeted inhibitors have been effectively created and exploited in prostate cancer clinical research. The peptidomimetic inhibitor EuK (lysine-urea-glutamate), a well-known PSMA inhibitor, was attached to hydroxy-nido-carborane to deliver boron to prostate cancer cells [147]. An analogous strategy was later employed by Wang et al., where one or two ortho-carborane molecules were linked to an alkyl spacer attached to the EuK inhibitor **20** (Figure 9).

Cell-penetrating peptides (CPPs) can cross cellular membranes, and their conjugation to carboranes resulted in an increased membrane crossing ability, leading to a significant boron accumulation in the cancer cells. Worthy of note is the recent synthesis of an ortho-carborane derivative **21** (Figure 9) obtained by the fusion with a short peptide, namely TAT (GRKKRRQRRRPQ) [151]. By adding hyaluronic acid to the solution, the authors generated a self-assembling micelle of negative surface charge able to shield the TAT from non-specific interactions during systemic circulation. The resulting carborane-TAT adduct exploited the cell-penetrating ability of TAT peptide to selectively enter and deliver boron to murine breast tumors up to ~20 mg/kg.

#### 2.1.8. Antibody Derivatives of Carboranes

**Figure 10:**
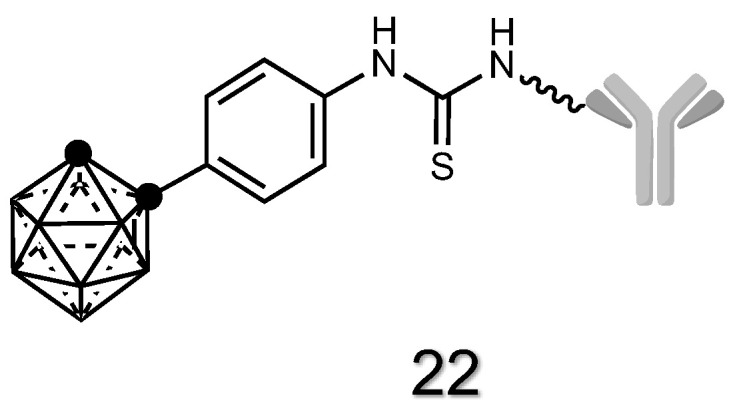
Structure of carborane-antibody conjugate (**22**).

Antibodies (Abs) are proteins that recognize and bind to specific proteins/receptors present on the cells and have a crucial role in the development of targeted therapies [153]. It is evident that the conjugation of fragments or full antibodies to carboranes may be used to develop targeted strategies for BNCT. Examples are mT84.66, Mu-9 or 107-1A4 antibodies [145,146,154,155]. The mT84.66 antibody is a murine monoclonal antibody that targets a specific antigen called carcinoembryonic antigen (CEA). CEA is a protein that is normally produced during fetal development, but its expression decreases after birth. However, certain types of cancer, including colorectal, gastric, pancreatic and lung cancers, can lead to the re-expression of CEA. Mu-9 **22** (Figure 10), a murine monoclonal antibody, specifically targets colon-specific antigen-p (CSAp), a tumor-associated antigen found in approximately 60% of colorectal carcinoma. 107-1A4, an IgG1 mAb, is selective for prostate cells [146].

### 2.2. Incorporation of Carboranes in Drug Delivery Systems for In Vivo Studies

Different carborane delivery systems were used for in vivo studies, which can be classified into three distinct categories: (i) supramolecular carriers, exploiting host–guest interactions, (ii) self-assembled supramolecular structures and (iii) conjugation at the surface or incorporation into nanoparticles.

These delivery systems present some common features, such as a high biocompatibility and solubility in physiological environments, but can differ in composition, size, morphology and delivery mechanism. Table S2 in the Supplementary Materials provides a comprehensive list of the drug delivery systems applied for carborane delivery during in vivo experiments.

#### 2.2.1. Supramolecular Carriers

**Figure 11:**
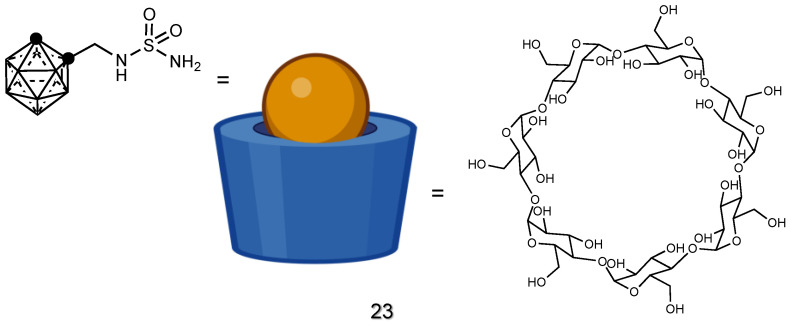
Structure of β-cyclodextrins and carborane sulfonamide (**23**) [110]. Image created with BioRender.com.

Cyclodextrins (CDs) can serve as carriers for carborane-based BDA [110], increasing the solubility of the drug by incapsulating it in their hydrophobic cavity. CDs are generally considered biocompatible and have a long history of use in pharmaceutical formulations. They have been extensively studied for safety, tolerability and compatibility with biological systems, making them suitable potential candidates for drug delivery applications. An example is the use of β-cyclodextrins to enhance the solubility of the sulfonamide derivative of carborane [110], as shown in **23** (Figure 11). Also, proteins can be considered supramolecular hosts for the delivery of hydrophobic moieties [156], behaving as “trojan horses” for theranostic applications [157]. The formation of supramolecular complexes between blood proteins and carboranes governs their pharmacokinetics and pharmacodynamics properties. Due to the presence of hydrophobic binding pockets, where endogenous or exogenous compounds can bind, proteins represent valuable delivery system platforms for carboranes [158,159].

#### 2.2.2. Self-Assembled Supramolecular Carriers

**Figure 12:**
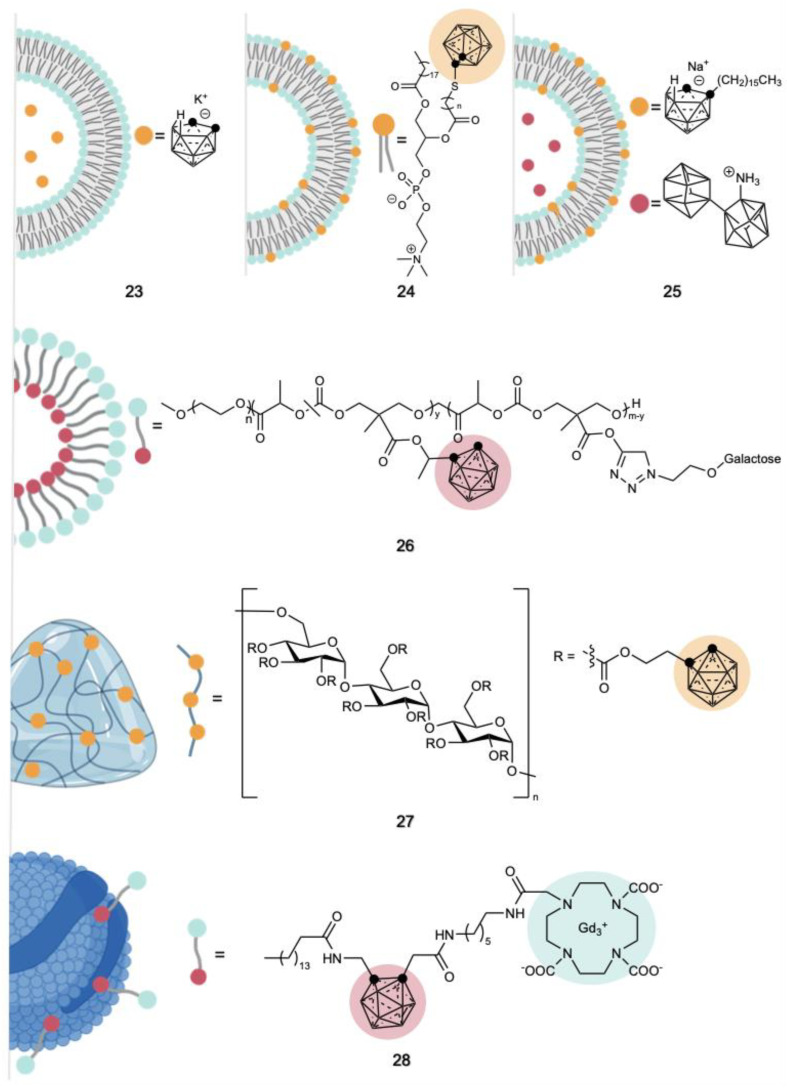
Schematic representation of carboranes incorporated into drug delivery systems such as liposomes (carborane in the core (**23**) [173], in the bilayer (**24**) [171] and in both compartments (**25**) [165]), micelle (**26**) [175], nanogel (**27**) [85] and LDL (**29**) [140]. Created with BioRender.com.

Liposomes are vesicular structures naturally formed by phospholipids dispersed in water and composed of bilayers that completely enclose an aqueous compartment within the lipid membrane. Depending on the chemical composition and functionalization, they can offer several advantages over conventional drug delivery systems, such as targeted delivery to specific sites and prolonged or regulated release of drugs. In addition, they contribute to the safeguarding of drugs from degradation and elimination, determining an enhanced therapeutic performance and reduced toxicity [160]. Two strategies are reported in the literature for the use of liposomes as carborane carriers for in vivo BNCT experiments: (i) the incorporation of the carborane in the lipid bilayer or (ii) the entrapment of the carborane cage in the aqueous core of the liposome. The earliest example of liposome application to carry carborane to cancer cells in vivo was reported in 1995 by Feaks, where distearoylphosphatidylcholine (DSPC) and cholesterol were used to incorporate the anionic [nido-7-CH_3_(CH_2_)_15_-7,8-C_2_B_9_H_11_]^−^ in the bilayer [161]. Later on, a mixture of a nido-carborane anion appended to two alkyl chains with DSPC and cholesterol was used to generate liposomes for in vivo carborane delivery to squamous carcinoma in hamsters [162,163]. The same liphophilic nido-carborane derivative has also been loaded into a transferrin PEG liposome [164] and used as part of the bilayer membrane used to incapsulate a borane molecule (TAC) in the hydrophilic core of the liposome, as shown in **25** (Figure 12) [165,166]. A vesicle structure bearing ortho-carborane-appended PEG was synthesized to enhance blood stability and cellular uptake [167]. In 2016, Takeuchi et al. were able to obtain a PEGylated liposome by exploiting 1-(4-methoxyphenyl)-1,7-nido-carborane with a C7 alkyl chain instead of cholesterol to generate the lipid membrane of the liposome with DSPC [168]. A biodegradable derivative of meta-carborane conjugated to poly(ethylene glycol) methyl ether (mPEG), which is amphiphilic and possesses self-assembling properties in water, was synthesized in 2016 [169]. An amide derivative of closo-carborane was also attached to an aliphatic chain that, together with PEG, generated a liposome containing 10 wt% of carborane [170]. A notable development of such a strategy is the recent design of boronsomes **24** (Figure 12) by Li et al. [171], where a carborane-appended phospholipid led to the formation of a biomimetic nanovesicle displaying high stability and boron delivery to murine breast tumors. In all these examples, the carboranes were dispersed within the bilayer membrane. However, a few studies reported their encapsulation into the liposome aqueous core. As an example, the hydrophilic 2-(dicarba-closo-dodecaborane) succinate was incorporated into liposomes obtained from a lipidic mixture of lecithin from egg yolk and cholesterol [172]. In a different study, Lee et al. used the water-soluble potassium salt of nido-7,8-carborane as a boron agent [173] and exploited the delivery ability of PEGylated liposomes **23** (Figure 12) to maximize the uptake into malignant cells while minimizing its presence in the reticuloendothelial system (RES).

Micelles, differing from liposomes due to the presence of a lipid monolayer instead of a bilayer, were also employed as carriers for the delivery of carborane for in vivo BNCT applications [174]. The addition of hyaluronic acid to the TAT-derivative of carborane enabled a self-assembling micelle of negative surface charge able to shield the TAT from non-specific interactions during systemic circulation [151]. As shown in **26** (Figure 12), a micelle composed of a carborane and MPEG was synthetized and tested in vivo in BNCT [175]. Another recent study [176] reported an AB-type Lactosome nanoparticle composed of amphipathic polydepsipeptide (i.e., polymers of α-hydroxy acids and α-amino acid [177]) linked to a hydrophilic polysarcosine (PSar) and a hydrophobic poly-L-lactic acid (PLLA). This resulted in self-assembling micelles that incorporated dihexyl-ortho-carborane and delivered it to breast cancer at a high boron concentration and with T/N ~ 3 and T/B > 5 after 72 h.

Nanogels, formed either through chemical crosslinking or physical self-assembly, demonstrated remarkable potential in encapsulating various types of therapeutics [178]. The nanoscale size of these carriers imparts them with distinct surface area and internal capacity, enhancing the stability of the enclosed drugs and extending their circulation duration. In 2017, pullulan, a polysaccharide consisting of repeating units of maltotriose, was derivatized with an ortho-carborane derivative, forming a cross-linked nanogel **27** (Figure 12) where carborane protrudes in the hydrophobic core [85].

Low density lipoproteins (LDL), which consist of an outer shell of phospholipids, cholesterol and apolipoproteins and a hydrophobic inner core, are widely employed delivery agents due to their high biocompatibility and targeting properties towards specific receptors. The lipid core of human plasma low-density lipoprotein (LDL) was extracted using hexane and the LDL was reconstituted with the addition of n-octyl-carborane [179]. LDL was also used to complex an ortho-carborane derivative bearing a crown-ether moiety where gadolinium (Gd^3+^) is entrapped, as shown in **28** (Figure 12) [140,141].

#### 2.2.3. Nanoparticles

An additional strategy applied to deliver carborane employed a polymeric self-assembled nanoparticle made of poly(ethylene glycol)-b-poly(L-lactide-co-2-methyl-2(2-dicarba-closo-dodecarborane)propyloxycarbonylpropyne carbonate) that encapsulates doxorubicin [180]. This system, based on an amphyphylic copolymer nanoparticle, aimed at performing a dual chemotherapy and BNCT treatments simultaneously and was tested in murine cervical cancer models. PLLGA nanoparticles are composed of lactic acid (LA) and glycolic acid (GA) monomers, which co-polymerize to obtain a biocompatible and biodegradable copolymer. In 2017, Takeuchi et al. [181] successfully loaded ortho-carborane into these nanoparticles, obtaining an adequate ^10^B tumor concentration of 20 mg/kg in B16 melanoma induced tumors. Furthermore, the tumor/blood ratios in their study exceeded 5, 8–12 h after the injection, suggesting that these nanoparticles could be effective carborane-based BDA for BNCT applications.

**Figure 13:**
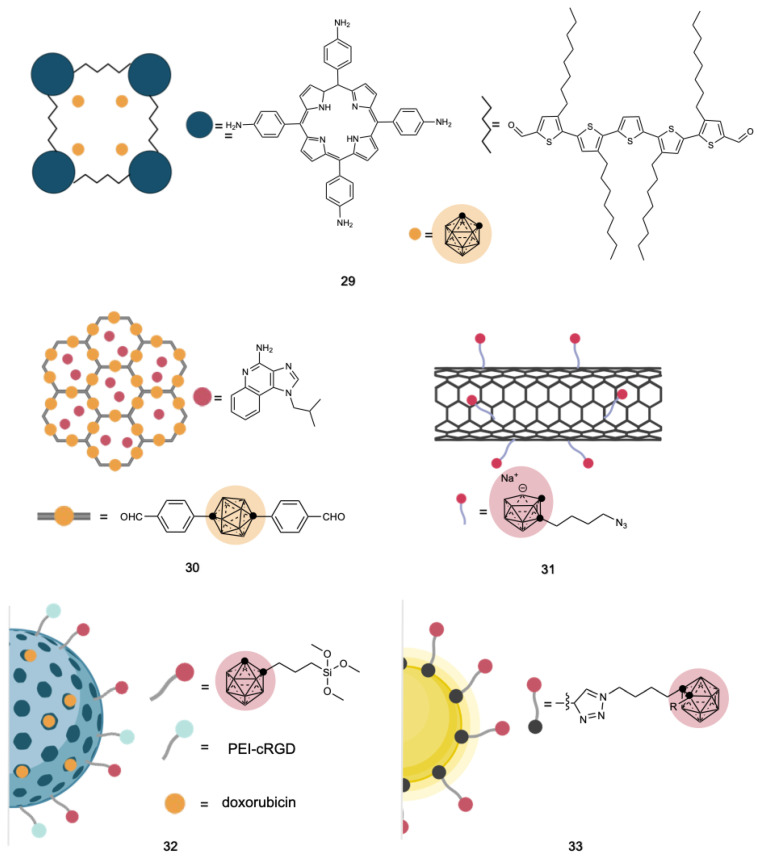
Schematic representation of carboranes incorporated into drug delivery systems such as COFs (porphyrin-based (**29**) [192] and carborane-based (**30**) [182]), SWCNTs (**31**) [183], HMSN (**32**) [185] and magnetic nanoparticles (**33**) [188]. Created with Bio.Render.com.

Covalent organic frameworks (COFs) are a class of porous, crystalline materials composed of organic molecules connected by covalent bonds. They possess a high degree of design flexibility, which allows for precise control of their structure and properties. A porphyrin-based polymer scaffold has been recently used to encapsulate pristine ortho-carborane to generate a BDA tested in vivo on a skin melanoma model **29** (Figure 13). In 2023, Shi et al. [182] reported a carborane-containing COF used as a capsule to deliver an immune adjuvant (imiquimod) upon localized nuclear irradiation **30** (Figure 13).

Carbon nanoparticles, such as single walled carbon nanotubes (SWCNTs), generally present high biocompatibility and low toxicity, making them suitable candidates for drug delivery applications. Due to their high surface area and easy functionalization, SWCNTs were derivatized with nido-carboranes (structure **31**, Figure 13), and the resulting conjugate displayed an enhanced boron accumulation in mammary carcinoma with respect to the healthy cells [183]. Graphene oxide was also grafted with a mono-iodinated COSAN derivative, obtaining a platform for traceable boron delivery through PET imaging [184].

Hollow mesoporous silica nanomaterials (HMSNs) are effective drug delivery systems for a variety of drugs, thanks to the high surface area and the possibility of tuning the pore size. Dendritic MSN decorated with PEG-cDRG and carborane moieties, and pored-loaded with doxorubicin, was synthetized in 2021 [185] **32** (Figure 13). HMSN was covalently modified with chitosan (CS), lactobionic (LA) and thioctic (TA) acids, which can target asialoglycoprotein receptors that are over-expressed in hepatocellular carcinomas [186]. Pristine ortho-carborane was added to the HMSN-CS-LA-TA composite, and BNCT in vivo experiments resulted in an effective treatment of hepatocellular carcinoma.

Magnetic nanoparticles (MNPs) have lately enlivened interest for their potential use in cancer therapy and targeted drug delivery [187]. These particles are obtained from metals or their mixtures, and their magnetic properties, under an external magnetic field, open to potential applications, especially in magnetic resonance imaging (MRI). In 2010, Zhu et al. reported the synthesis of carborane-containing magnetic nano-composites obtained by the “click-reaction” between propargyl group-enriched magnetic nanoparticles and an azide derivative of ortho-carborane **33** (Figure 13) [188].

Gold nanoparticles were also functionalized with amine-nido-carboranes to enhance the boron accumulation in cervical cancer cells [189]. In particular, the fluorescent properties of gold nanoclusters allowed for the precise bioimaging of cancer cells and the targeted delivery of a carborane compound to the HeLa induced tumors. Recently, Pulagam and colleagues tested Au nanoparticles [190] and Au nanorods [191] to vehiculate boron to cancer cells by covalently attaching COSAN derivatives to the metal particles. The gold particles showed good in vivo stability but poor accumulation in the targeted tissues. The authors suggested that the performance of such conjugates may be enhanced by appropriate modifications of the gold core size and shape.

### 2.3. Formulation and Administration of Carboranes for In Vivo Studies

The synthesized carborane derivatives/carborane delivery systems were administered to mice by either (i) the direct injection of the developed chemicals dispersed in water (or physiological buffer such as PBS) or by using modifiers, enhancing their solubility, such as (ii) organic solvent or (iii) formulants (Supplementary Materials, Table S3).

Only in the work of Vincente et al. [125] was the use of water as the sole solvent (clearly) reported: in this particular formulation, the presence of nido-carboranes, negatively charged, probably sufficed for the hydrophilicity of the whole molecule. Aqueous solutions such as phosphate buffer saline (PBS) are desirable for injection due to high biocompatibility and have been generally used in the carborane-based formulations incorporating liposomes and micelle [113,135,161,168,172,173,175].

Organic solvents are occasionally employed in drug administration to allow the solubilization of hydrophobic drugs. In general, large fractions of organic solvents are not desirable in the final formulation to be tested because of the intrinsic toxicity and side effects that may arise from their presence. It must be noted that, given the low water solubility of carboranes, basically all the synthetic pathways involved the use of solvents such as ethanol and DMSO. However, most of these solvents are removed during sample processing and may only be present in traces in the final formula. Nonetheless, some final carborane conjugates required a small percentage of such solvents to enhance the solubility before administration. In particular, dimethylsulfoxide (DMSO), ethanol (EtOH) or chloroform (CHCl_3_) have been employed in variable amounts to dissolve and administer carborane derivatives with nucleosides [90,92,94,95,96,97,193], porphyrines [132,133], nitroimidazoles [108], SWCNTs [183] and graphene oxide [184].

Formulants such as cremophor EL (CrEL) obtained by the reaction of ethylene oxide and castor oil are non-ionic solubilizers and emulsifiers and have been extensively used for the administration of poorly water-soluble drugs. CrEL has been used from the earliest works of Miura [114,115,116,117,118,119,121,122,124,126,131] as a solubilizer for porphyrin-based carborane drugs, together with propylene glycol. Despite its broad use, CrEl has been reported to be not inert and can potentially induce some clinical reactions [194].

### 2.4. Cancer Models Used for In Vivo Studies Using Carboranes in BNCT

The in vivo experiments for testing BNCT are typically performed on mice (only a recent work by Ferrer-Ugalde [184] used *C. elegans* nematodes). Here, in some cases (12% of the studies) the carborane formulations were injected in non-tumoral models in order to monitor only endpoints such as bio-distribution and drug toxicity [103,167,195]. However, in the majority of the cases, a tumor was induced in mice by the subcutaneous implantation of malignant cells in order to study tissue bioaccumulation as well as the efficiency of the carborane-based BNCT treatment on mice survival and tumor reduction (Supplementary Materials, Table S4).

Cancer lines studied were either from rodent (67% of the studies) or human (21% of the studies) lines. In general, the most-studied tumor types were breast (4T1, BT-474, BCAP-37, EMT6, Her2+ and KHJJ lines) and brain cancers (9LGS, C6, F98, GL261, RG2, U87 and U373 lines), investigated in 22% and 19% of the studies, respectively. Other works mainly focused on skin (B16, Harding-Passey melanoma, MM-138 lines, 15% of the studies), connective tissues (AB22, KHT, L929, M-1 sarcoma, SCCVII, 12% of the studies) and colon (ARO, Colon-26, CT26, GW-39, LS-174T lines, 8% of the studies). Cancer lines investigated to a lesser extent included prostate, liver, pancreas and reproductive system cancers. A list of the studies classified by cancer type, cell lines tested to induce tumors and murine models is reported in the Supplementary Materials, Table S4.

## 3. Analytical Methodologies Used for Carborane Quantification in In Vivo Studies

The possibility to carry out effective BNCT necessarily relies on the delivery of B to cancer cells at concentrations in the range of 20–40 µg [^10^B]/g [tumor] [17], corresponding to approximately 10^9^ atoms of ^10^B per cell [196].

The right choice of the derivatization or the use of an appropriate delivery system for carboranes led to better intra-tumoral boron concentrations compared to BPA or BSH, reaching values higher than 50 ppm [86,94,115,118,134,135,171,175]. In some cases, the concentration was even greater than 100 ppm [86,135,175].

Intracellular localization of the carborane is necessary for an effective BNCT therapy. In general, cellular uptake of carboranes is more complicated than BPA, due to their lack of a cell-specific import system. However, the use of targeting agents or effective delivery systems in many cases determined the expected cellular uptake of the carboranes [84,86,94,105,134,139,175,186].

The quantification of ^10^B concentrations is crucial for BNCT to define (i) an effective treatment window and (ii) the appropriate neutron fluence rate. Accurate and real-time assessment of tumor boron concentration in the cancer tissue determines the efficiency of the BNCT treatment. Ideal treatments require the selective accumulation in tumors as well as a low toxicity and a functional residence time and metabolism of the injected drugs.

Some of the developed carborane derivatives showed a better pharmacokinetic and pharmacodynamic profile (i.e., tumor accumulation time) than BSH and BPA [83,94,118,137,139,175,186].

Accordingly, many papers determined B/carborane concentrations and biodistribution in order to define bioaccumulation and partitioning in mice tumoral models. Typical endpoints investigated include the quantitation of B in tumor, surrounding tissues, biological fluids and their ratios as well as the imaging and monitoring of boron at organ, tissue and (sub)cellular levels.

The methodologies applied for B analysis in BNCT have been extensively reviewed by Wittig et al. [197], and, more recently, by Dai et al. [198]. Here, we discuss the techniques employed during in vivo studies focused on carborane-based drugs. A main distinction among the different techniques can be made between (i) methodologies aiming at the quantification of the boron amount in the samples and (ii) those focusing on carborane localization/quantification by measuring the specific signal provided by imaging tags attached to the carborane cage.

In the first case, methodologies such as prompt gamma-ray neutron activation analysis (PGAA) and optical emission spectroscopy (OES), often referred as atomic emission spectroscopy (AES), were the most widely used techniques. Here, biodistribution was assessed by ex vivo analysis of mice tissues and biological fluids. In the second case, the functionalization of the carboranes with fluorophores, contrast agents or radionuclides allowed ex vivo analysis but also the application of in vivo methodologies such as fluorescence imaging, MRI or tomography-based techniques. The following chapter briefly summarizes the concepts underlying each methodology, whereas comprehensive tables summarizing the studies employing them are provided in the Supplementary Materials.

### 3.1. Gamma-Ray Measurements

**Figure 14:**
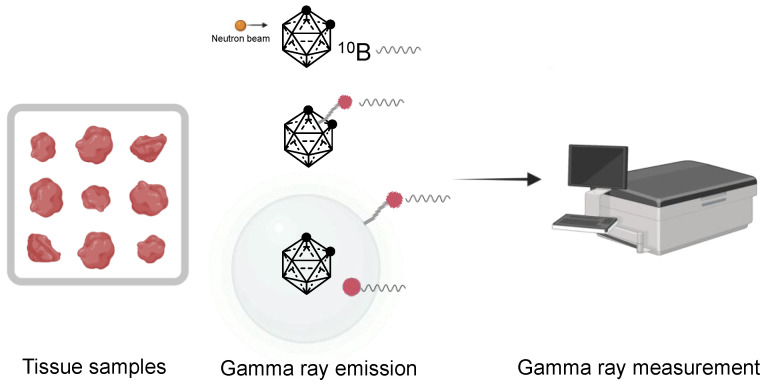
Schematic representation of the techniques for carborane detection using gamma-ray emission measurements. Red circle represents the radionuclides. Created with BioRender.com.

The quantification of carboranes in samples from in vivo experiments was often achieved by quantifying gamma-ray emission in the samples. It must be noted that natural boron isotopes are not radionuclides. Hence, detection was achieved based on two different strategies: (i) the irradiation of carboranes with a beam of neutron and the spectrometric measurement of the resulting prompt-gamma ray emitted or (ii) the labeling of carboranes with gamma-ray emitters followed by gamma-counter analysis (Figure 14).

#### 3.1.1. Prompt Gamma-Ray Neutron Activation Analysis (PGAA)

PGAA is a widely used technique exploiting the same principle of the BNCT reaction for the detection of B in solid or liquid samples. The measurement relies on the spectrometric detection of prompt gamma neutrons emitted by ^10^B upon irradiation with a thermal neutron beam [199]. The gamma ray energies allow B identification, whereas their intensities allow its quantitation. In carborane studies, PGAA was routinely applied to the characterization of B concentrations in the samples collected from in vivo tests as well as to the characterization of the carborane formulation prior to injection (Supplementary Materials, Table S5). Such a methodology presents the advantages of being relatively fast, with measurements achieved in the range of a few minutes for B concentrations in the ppm range. In addition, sample preparation is straightforward, and the analysis is non-destructive, with potential clinical application, i.e., for B concentration screening in patients during BNCT treatment [197]. A further advantage of PGAA is that the detection measures the ^10^B isotope over the total B load. However, limitations are linked to the relatively low sensitivity compared to other techniques and the need for dedicated instruments and facilities.

#### 3.1.2. Carborane Labeling with Gamma-Ray Emitters

Gamma-ray emitters such as ^64^Cu, ^68^Ga, ^125^I, ^211^At can be chemically incorporated to the carborane cage or to the carborane formulation. In many studies, radioiodination reactions were carried out to covalently bind ^125^I to B atoms in the carborane clusters such as COSAN, FESAN or nido-carborane [16,143,144]. Here, the iodine tracer was used to visualize the bioaccumulation in tumors and organs but also to confirm the stability of the novel drugs, i.e., by measuring the (absence of) I radioactivity in the thyroid glands.

Interestingly, Gona et al. [143] used this strategy to label Pegylated COSAN with either ^124^I (positron emitter) or ^125^I (gamma emitter), which allowed biodistribution analysis by in vivo PET-CT (described below) and ex vivo gamma counter, respectively. The authors reported a good correlation between the two sets of results and highlighted the advantages of a multi-technique approach for a better quantitation of B bioaccumulation and distribution. A similar approach was applied more recently by Pulagam and colleagues [191], which labeled COSAN-derivatized Au nanoparticles incorporating ^64^Cu. Here, ex vivo (Cu-based) gamma counter in the mice organs/tissues was combined with other chemical analysis and ^64^Cu-PET-CT to provide accurate biodistribution data in nude mice xenografts.

In general, the ex vivo quantitation by gamma-counter analysis was extensively applied, especially in pioneering studies, with the use of carboranes labeled with ^125^I but also other radioactive species such as ^211^At and ^68^Ga. A list of the studies employing carborane radio-labeling/gamma counter analysis is reported in the Supplementary Materials (Table S6).

### 3.2. Boron Elemental Analysis Techniques

**Figure 15:**
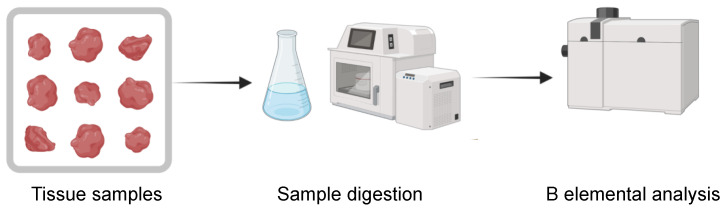
Schematic representation of sample processing and measurements for boron detection using boron elemental analysis techniques. Created with BioRender.com.

Elemental analysis provides information about the elemental composition of a sample, which in the case of carboranes focuses on boron detection. Most of the in vivo studies determined the B concentration ex vivo by spectroscopic OES measurements. Here, the detection is achieved by measuring the radiation wavelengths emitted by a given element upon excitement in a plasma, which can be generated by several methods, i.e., Direct Current Plasma (DCP-OES), Microwave (MW-OES) and Inductively Coupled Plasma (ICP-OES) [200]. Instruments such as ICP-OES are widely available in laboratories worldwide and often represent the analytical method of choice for routine analysis of metals and metalloids. However, AES analyses are limited by a relatively high detection limit (ppm range) and cannot discriminate between B isotopes. This is particularly relevant considering that the ^10^B isotope, needed for BNCT reaction, accounts for only approximately 20% of the natural boron abundance. It is worth noticing that some studies tested ^10^B-enriched carborane, which can increase BNCT performance and inherently facilitate ^10^B detection [97,138,174]. However, a better B analysis was achieved with Inductively Coupled Plasma Mass Spectrometry (ICP-MS), where mass spectrometric detection granted a more sensitive B analysis (ppb range) and a higher selectivity for the ^10^B species of interest [84,186]. In general, analyses by ICP-AES and ICP-MS were carried out in up to 64% of the in vivo studies considered herein (Supplementary Materials, Table S7). Usually, carborane formulations as well as biological samples were treated prior to analysis with a combination of strong acids (e.g., nitric acid, sulfuric acid) and oxidants (e.g., hydrogen peroxide) to fully disrupt both carborane structures and biological matrices and to homogenize the samples (Figure 15).

In the case of more complex samples such as mice tissues or even organs, a better digestion was achieved with perchloric acid and treatments at relatively high temperatures. Microwave-assisted digestion was also employed for assuring a complete digestion of such samples [110,165,200]. Recent advances include the combination of acid digestion and UV irradiation for the ICP-MS detection of boron in biological matrices [201]. Such methodologies allowed L-BPA detection in cell cultured in vitro at levels below ppt, and they are also promising for future analysis of in vivo experiment samples and carborane molecules.

It must be noted that ICP-AES and ICP-MS analysis can be challenging due to the peculiar properties and behavior of B species that can affect proper qualitative and quantitative determination, especially in complex matrices. These include a strong memory and carry-over effects during the measurements as well as the formation of volatile B compounds. Such problematics have been extensively discussed elsewhere [200] and should be carefully considered during the experiments. In addition, matrix components can result in a reduced B extraction, high background elements and signal suppression. In a few instances, an internal standard, such as Berillium [179], Rhodium [200], Yttrium [126] or Lithium [163], was added to the samples prior to digestion for a better quantification of the boron load.

A drawback in the analyses by techniques such as PGAA and ICP-AES is that they are not selective for carborane and reveal the boron content independently of its state. In addition, although very robust for determining samples’ concentration in homogeneous samples at macroscopic levels (tissues or even organs), such techniques cannot provide information about partitioning and inhomogeneities at the micro (sub)cellular scale. Furthermore, plasma-based analysis is destructive, which should be considered if further observations are planned for the samples under investigation.

### 3.3. Fluorescence-Spectroscopy/Microscopy

Although pristine carboranes do not display typical absorption and emission properties, in the last decade fluorescence detection has been widely used for their quantification/imaging by functionalizing their structures with fluorescent dyes. The resulting labeled structures allowed the quantitation of carboranes at low ppm levels and their localization with micrometric resolution.

Functionalization was achieved with the chemical conjugation of the dye to the carborane [122,132] or its incorporation in carborane-supramolecular assemblies [175,185]. In the first case, the conjugates obtained by the covalent binding of the carboranes with porphyrins were observed to be stable in vivo [132] and could be monitored in whole organs and tissues as well as at intra-cellular levels. Porphyrins and phthalocyanines typically emit light in the 600–800 nm range, which allows a sensitive detection in complex organic samples and an estimation of drug bioaccumulation and stability by ex vivo analysis of treated mice samples at specific time intervals. Furthermore, given the covalent bond stability, the B concentration could be calculated on the basis of the stechiometry between porphyrines and carboranes in the injected formulation [122,132,134,139].

Other studies employed fluorescent dyes that were not necessarily bound to the carboranes but incorporated in the developed formulation. For instance, Rhodamine-B was used to label carborane-containing supramolecular assemblies such as micelle [169,175], liposomes [105,167] and nanogel [85]. Similarly, Doxorubicin was introduced as an anticancer drug and fluorescent tracer in carborane-based mesoporous [185], vesicles and polymeric nanoparticles [169,202]. Many different dyes were employed for studying tumor distribution and cellular localization of carborane derivatives, including carbocyanine-based (e.g., DiR, DiI, DiO), Cyanine-5.5 (Cy5.5) [84,202], Indocyanine Green (ICG) [176] and VivoTrack 680 for liposomes labeling [173]. Wang et al. [202] physically incorporated carbocyanine-based fluorescent probes into boron-containing vescicles (BCVs) as donor–acceptor pairs for fluorescence resonance energy transfer (FRET). A summary of the studies employing fluorescence-based techniques for carborane analysis during in vivo research is provided in the Supplementary Materials (Table S8). It must be noted that in these works, the quantitation of carborane by fluorescence measurements was often confirmed by additional analysis such as ICP-AES or ICP-MS [180,185].

**Figure 16:**
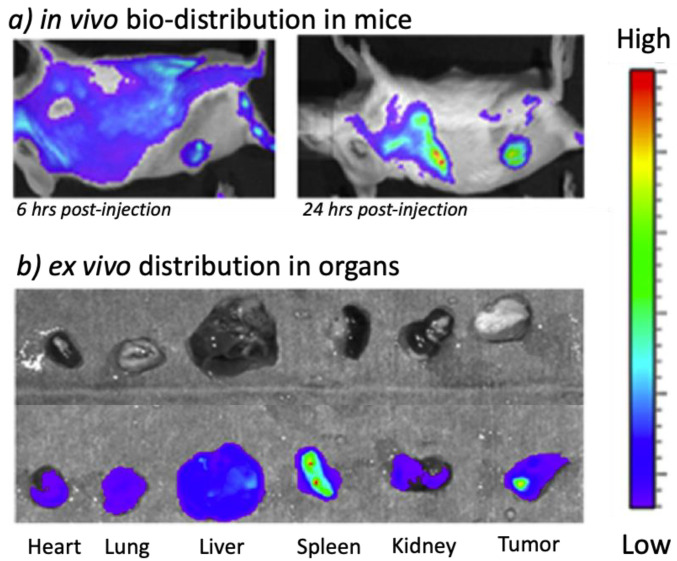
Fluorescence-based analysis of carborane-bearing pullulan nanogels incorporating rhodamine-b as fluorescent dye. (**a**) The in-vivo biodistribution in mice 6 h (left) and 24 h (right) after injection of the carborane drug. (**b**) The ex vivo analysis of the tumor in organs and tumor of controls (top) and carborane-treated mice (bottom). Reprinted from ref [85] with permission from Elsevier.

It is also noteworthy that most studies discussed so far performed fluorescence imaging analysis ex vivo. However, this technique offers the possibility for real-time in vivo imaging, which could be an added value for imaging guided treatments dosing and monitoring in BNCT experiments (Figure 16). As an example, Wang et al. [189] performed the in vivo monitoring of fluorescent Au nanoparticles (GNCs) incorporating a carborane derivative. This granted both an accurate tumor localization/imaging and real-time bioaccumulation data in cervical cancer induced in nude mice. Similarly, in recent studies, DiR-capsulated nanoparticles, Cy5.5-modified nanoparticles and ICG-labeled lactosomes were used to visualize the biodistribution in ex vivo organs but also for real-time in vivo monitoring of the carborane drugs up to 72 h after injection [84,176].

### 3.4. PET/SPECT

**Figure 17:**
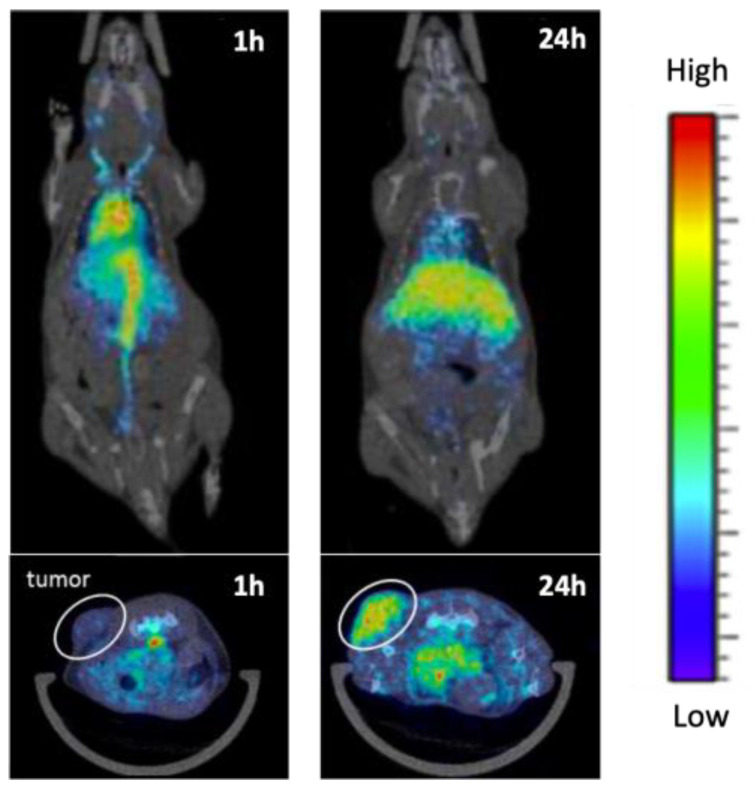
An example of in vivo PET analysis of murine tumoral model after injection of COSAN-conjugated [^64^Cu]-enriched AuNPs at different time intervals. [^64^Cu]-based detection allowed imaging of the tumor morphology and localization of the carborane-based drug. Reprinted from ref [203] with permission from John Wiley and Sons.

The in vivo spatial distribution of BNCT carborane-based chemicals was also achieved with tomographic techniques such as single-photon emission computed tomography (SPECT) and positron emission tomography (PET). In the last decade, the latter has emerged as a reference technique for monitoring drug delivery and tumor development, especially in combination with X-ray computed tomography (CT). PET-CT analysis allows for the simultaneous determination of drug biodistribution and pharmacokinetics together with tumor volume and morphology (Figure 17). The analysis is performed by measuring positrons emitted by marked radioisotopes associated with the drugs, and it provides both qualitative 3D biodistribution and quantitative data, e.g., expressed as the fraction of the injected dose per mass or volume (%ID/cm^3^). As shown in Table S9 (Supplementary Materials), although tomography-based techniques are regarded as some of the best-performing approaches for studying cancer systems in vivo, their use in carborane-BNCT studies is relatively limited. ^64^Cu was used as radiotracer in recent works focused on carboranes conjugated with Au nanoparticles [203], nanorods [191], nanoscale covalent organic polymers (COPs) [192] and boronsome, innovative carborane-based liposomes showing high stability, tumor bioaccumulation and residency [171]. Here, the radiotracer was physically trapped into the supramolecular assembly containing the carboranes. ^124^I was also used as a PET tracer for studying the biodistribution of carborane-doped graphene oxide sheets, further functionalized with iodine [184]. Interestingly, a study on COSAN-PEGilated gold nanoparticles incorporated the ^124^I tracer either at the core or at the shell of the nano-structures, and it performed a dual “core-shell” analysis to show the integrity of the developed drug after injection in mice [190]. Recent studies also employed less common radioisotopes such as ^68^Ga and ^89^Zr. In 2019, Wang et al. [152] bound ^68^Ga radioisotopes to a PSMA inhibitor conjugated to carboranes and combined in vivo (Ga-based) PET with ex vivo ICP-AES analysis for drug biodistribution assessment. ^89^Zr was also incorporated into the carborane-based covalent organic framework (B-COF), resulting in an optimal detection by PET-CT analysis in tumor-bearing mice [182].

SPECT was reported in a study from Genady et al. [144] to assess the biodistribution in nude mice xenografts injected with carborane derivatives and labeled with iodine radionuclides. Here, iodocarboranyl tetrazine was alternatively labeled with ^125^I or ^123^I for gamma counter and animal imaging, respectively.

### 3.5. Magnetic Resonance Imaging (MRI)

**Figure 18:**
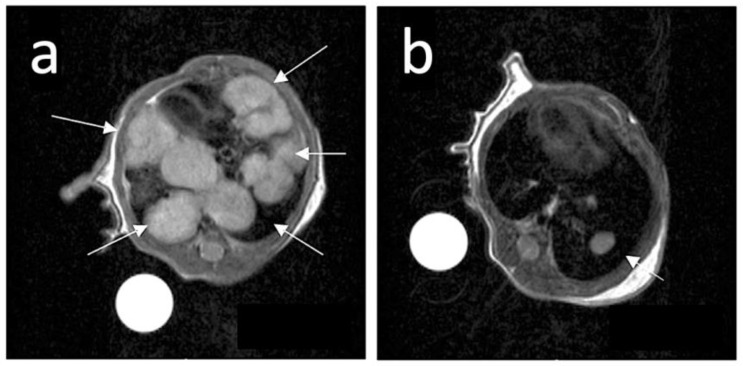
Magnetic resonance imaging (MRI) analysis of liver metastases in Balb/c mice. The arrows identify the tumor in non-treated (**a**) and Gd-carboranes conjugate-treated (**b**) samples after in vivo BNCT treatment. Reprinted from ref [141] with permission from Elsevier.

Similar to PET, magnetic resonance imaging (MRI) is a powerful imaging technique which can provide non-invasive, in vivo mapping data valuable for clinical studies (Figure 18). Although the detection of ^10^B and ^11^B by MRI is challenging, such methodology has been optimized for BSH and BPA compounds in recent years [198]. However, in the case of carboranes, only a few studies employed MRI (Supplementary Materials, Table S10). A first work from Wood et al. [108] focused on the in vivo analysis of ^11^B MR spectra after mice injection with nitroimidazole-carborane, resulting in a proper detection of carboranes in the tumor and organs but no imaging of the cancer. More recently, other studies used a Gd-tracer, which is often used in MRI as a contrast agent [140,141]. Two studies combined Gd to ortho-carboranes and enhanced the dispersion with β-cyclodextrin and low-density lipoproteins. The resulting carborane conjugates combined the possibility of delivering high boron quantities to cancer cells while allowing for in vivo monitoring of tumor morphology, growth and development (Figure 5). A similar strategy was previously tested by Nakamura et al. [142], who covalently bound ortho-carborane to a commercially available Gd-DTPA complex contrast agent (Magnevist^®^), which was dissolved in NaHCO_3_ solution and directly injected in mice. Here, carborane biodistribution was assessed with the combination of in vivo (Gd-based) MRI detection with ex vivo analysis by ICP-MS and alpha-radiography.

### 3.6. Other Methodologies for Carborane In Vivo Analysis

It must be noted that several techniques widely applied in BNCT research with BPA and BSH compounds are in principle valid for carborane analysis but were seldom or not employed in the studies reported here. For instance, methodologies such as quantitative neutron capture radiography (QNCR) and secondary ion mass spectrometry (SIMS) found application in pioneering carborane-based works but were not routinely applied for in vivo BNCT studies [113,200,204]. Radio high-performance liquid chromatography (HPLC) was employed in two studies by Schinazi et al. for the analysis of carborane-functionalized radio-labeled nucleosides [92,93]. Similarly, established alpha-radiography methods for B analysis in BNCT experiments were seldom applied during in vivo carborane studies [131,142]. Only two studies [130,190] used (micro) Particle Induced X-ray Emission (μ -PIXE) for the determination of the ex vivo micro-distribution of either Cu-porphyrin-carboranes or I-labeled carborane-gold cluster [130]. In particular, Pulagam et al. [190] combined μPIXE with micro-Rutherford backscattering (μRBS) to simultaneously study the spatial distribution of elements originated from tissue and NPs collected in vivo. The results were then combined with previously acquired PET-CT data collected from the same animals. Also, in a later study, the same authors combined multiple techniques for in vivo (PET-CT) and ex vivo (ICP-MS, gamma counter) analysis of mice bearing gastroinstestinal cancer [191]. Given the complexity of the in vivo BNCT experiments, and the need to collect multiple endpoints (e.g., time-resolved carborane concentration and tumor localization/development), similar strategies based on multiple detection techniques are desirable, and should be considered in future works.

To conclude, other methodologies, such as nuclear magnetic resonance (NMR) and HPLC coupled to UV or MS detection, were applied during carborane drug synthesis, formulation and purification, but not for in vivo samples analysis [90,147,205]. Similarly, transmission electron microscopy (TEM) was used to characterize synthesized carborane supramolecular structures, but only recently to assess the in vivo cyto-distribution of boron capsules in tumor tissues [182]. It is noteworthy that such techniques can provide accurate qualitative/quantitative data during preparatory analysis and characterization and could be exploited by future studies for a better understanding of carborane drug pharmacokinetics, metabolism and transformation during treatments.

## 4. Conclusions

Boron neutron capture therapy (BNCT) is an emerging anticancer modality and carboranes are among the most promising boron agents for BNCT.

In this review, we performed a screening of the existing literature on the topic and focused on in vivo studies, which, although representing only a small portion of the available literature on carboranes, are the real test-beds for clinical translation.

In general, few studies employed pristine carboranes as boron agents due to their hydrophobicity, which hampers their direct in vivo administration. On the other hand, many carborane derivatives were synthesized to overcome their low water solubility. Derivatization of the carborane cage usually improves the pharmacokinetic, pharmacodynamic and physicochemical properties of the molecule. Additionally, some moieties (i.e., porphyrins, carbohydrates or small peptides) may improve cancer cell selectivity and absorption.

Alternatively, delivery systems such as supramolecular carriers, self-assembled supramolecular structures or nanoparticles were used to carry carboranes in physiological environments for in vivo studies. Although they share several characteristics, such as a high biocompatibility and solubility in physiological settings, these delivery systems can vary in content, size, shape and delivery method.

The ideal solution to administer the synthesized carborane-derivatives/carborane-delivery systems is water (or a physiological buffer such as PBS); however, some formulations required small percentages of organic solvents or formulants to effectively solubilize the carborane. The in vivo experiments for testing BNCT are typically performed on mice, where murine (67%) or human cancer cells (21%) are implanted. The most-studied tumor types were breast and brain cancers.

The delivery of sufficient amounts of ^10^B to tumor cells is essential for the efficacy of BNCT treatment. With regard to the qualitative and quantitative assessment of boron biodistribution upon in vivo drug administration, most of the studies we examined employed plasma-based methodologies, such as DCP- and ICP-AES. Such techniques are widely available and allow a reliable and fast boron analysis, but require extensive sample treatments (i.e., samples acid digestion) and provide biodistribution data exclusively ex vivo. Several studies incorporated fluorescent dyes or exploited the fluorescence properties of carboranes conjugates in order to carry out spectroscopic analysis targeting the drugs. This approach allowed a real-time in vivo analysis of drug biodistribution by fluorescence microscopy. Nonetheless, other powerful techniques for in vivo screening, such as MRI and PET-CT, were applied to a lesser extent and should be considered in future works. In particular, when assessing multiple endpoints (e.g., boron delivery/quantitation, tumor localization/development), the best results were often obtained by combining multiple methodologies, for instance imaging techniques (fluorescence or PET) with chemical analysis such as ICP-AES.

Imaging tags can be attached to the carborane cage or included in the delivery system to create innovative theranostic platforms. Drugs or photosensitizers are also used in conjunction with carboranes to develop a synergistic action between the BNCT treatment and chemotherapy/photodynamic therapy. Receptor-targeted BNCT or the use of therapeutic nucleic acids [204,206,207,208,209] may represent innovative approaches to improve the selectivity of boron agents. Biocompatible delivery systems, based on biomolecules such as proteins (i.e., albumin) or antibodies, will allow for better bioretention and bioavailability of the investigated boron agents [104,158,210,211,212,213,214,215].

In summary, carboranes display great potential for the development of next-generation BNCT drugs. The optimization of carborane conjugations with novel functional groups or delivery systems allows high boron concentration and tumor selectivity as well as an accurate screening of drug delivery and tumor localization in mice.

## Data Availability

No new data were created in this study. Data sharing is not applicable to this article.

## Supplementary Materials

The following supporting information can be downloaded at: https://www.mdpi.com/article/10.3390/cancers15204944/s1, Table S1: list of the carborane derivatives tested in vivo in BNCT; Table S2: list of the drug delivery systems tested in vivo in BNCT; Table S3: formulation used for the administration of the carborane-based drug in in vivo BNCT; Table S4: classification of the in vivo BNCT studies based on the type of cancer investigated, cell line used and its origin; Table S5: in vivo BNCT carborane studies employing prompt gamma-ray neutron activation analysis (PGAA) for the analysis of boron content; Table S6: in vivo BNCT carborane studies employing gamma-ray emission analysis for the detection of radionuclides-labeled carborane conjugates; Table S7: in vivo BNCT carborane studies employing plasma-based techniques for the (ex vivo) elemental analysis of boron. Table S8: in vivo BNCT carborane studies employing fluorescence-based techniques for the detection of carborane conjugates; Table S9: in vivo BNCT studies employing tomography-based techniques for the imaging of the carborane drugs; Table S10: in vivo BNCT studies employing magnetic resonance imaging (MRI) for the imaging of the carborane drugs.
