# Supplementary material for: In Vivo Application of Carboranes for Boron Neutron Capture Therapy (BNCT): Structure, Formulation and Analytical Methods for Detection
Source: Cancers (Basel). 2023 Oct 11;15(20):4944. doi: 10.3390/cancers15204944 (PMC10605826; doi:10.3390/cancers15204944)
Supplement: Supplementary file 1 [file cancers-15-04944-s001.zip › cancers-2561814-supplementary.pdf]

Supplementary Materials

In vivo application of carboranes for Boron Neutron Capture Therapy (BNCT): structure, formulation and analytical methods for carborane detection.

Tainah Dorina Marforio, Andrea Carboni* and Matteo Calvaresi*

Dipartimento di Chimica "Giacomo Ciamician", Alma Mater Studiorum—Università di Bologna, Via Francesco Selmi 2, 40126 Bologna, Italy

* Correspondence: andrea.carboni9@unibo.it (A. C.); matteo.calvaresi3@unibo.it (M.C.)

Abbreviations used in the Supplementary Materials tables:

Au-NP: Gold Nanoparticles
B-CD: Beta-cyclodextrin
BOPP: Boronated porphyrin
CD: Carborane Derivatives
DCP: Direct current plasma
COP: Covalent organic polymers
cRGD: Cyclic Arg-Gly-Asp peptide
$CHCl_3$: Chloroform
COF: Covalent Organic Framework
COSAN: Dicarbollylcobaltate
CRM: polyethoxylated castor oil (Cromophor EL)
Cy5.5: Sulfo-cyanine N-Hydroxysuccinimide esther
DDS: Drug Delivery Agent
DiR: 1,1′-dioctadecyl-3,3,3′,3′-tetramethylindotricarbocyanine iodide
DMSO: Dimethyl sulfoxide
DMF: Dimethylformamide
DPPC: 1,2-dipalmitoyl-sn-glycero-3-phosphocholine
DPPG: 1,2-dihexadecanoyl-sn-glycero-3-phospho- (10 -rac-glycerol)
DSCP: Distearoylphosphatidylcholine
DSPE-PEG: 1,2-distearoyl-sn-glycero-3-phosphoethanolamine-N-[amino(polyethylene glycol)
Egg PC: L-α-phosphatidylcholine
EtOH: Ethanol
F(Ab')-NEM: N- Ethylmaleimide bound antibody
GNC: Gold Nano Cluster
ICP: Inductively coupled plasma
IRGD: Cyclic 9-amino acid peptide (sequence: CRGDKGPDC)
LDL: Low-density lipoprotein
mAb: Monoclonal antibody
MPEG: Methoxypoly(ethylene glycol)
n.a.: not available
n.s.: not specified
OES: Optical Emission Spectroscopy
PBS: Phospate buffer saline
PDNA: Plasmid DNA
PEG: Polyethylene glycol
PLLA: Poly-L-lactic acid
PLGA / PLLGA: Poly(L-Lactide-co-Glycolide)
PLMB: PEG-b-P(LA-co-MPCB)
PRG: Propylene Glycole

PGAA: Prompt gamma-ray neutron activation analysis
PSMA: prostate-specific membrane antigen
SWCNT: Single-walled carbon nanotubes
TAT: Transactivator of transcription peptide (GRKKRRQRRRPQ)
TCPH: tetracarboranylphenyl porphyrin core (e.g. H2TCP, CuTCPH, ZnTCPH)
TPFC: Tetrakis(p-Carboranylthio-Tetrafluorophenyl)Chlorin
VCDP: 2,4-divinyl-nido-o-carboranyl-deuteroporphyrin IX

Tabls S1. List of the carborane derivatives tested in in vivo BNCT

Derivative Category	Derivative Moiety	Carborane Core	Ref.
carbohydrate	Maltoside	ortho-closo-carborane	[1]
	Chitosan	ortho-closo-carborane	[2]
	Pullulan	ortho-closo-carborane	[3,4]
nucleoside	Deoxyuridine	ortho-closo-carborane	[5]<br>[6]<br>[7]<br>[8]
	Timidine (N5-2OH)	ortho-closo-carborane	[9],[10],[11],[12],[13]
		meta-nido-carborane	[14],[15]
	Uracil	ortho-closo-carborane	[16]
	Pyrimidine (Nucleoside analogue)	ortho-nido-carborane	[17]
Drug	Sunitinib	ortho-closo-carborane	[18]
	Erlotinib	ortho-closo-carborane	[19]
	Nitroimidazole	nido-carborane	[20]
		ortho-closo-carborane	[21]
	Sulfonamide	ortho-closo-carborane	[22]
	Doxorubicin	ortho-closo-carborane	[23]
Porphyrin	CuTCPH	ortho-closo-carborane	[24],[25],[26],[27],[28],<br>[29],[30],[31],[32]
	VCDP	nido-closo-carborane	[33]
	ZnTCPH	ortho-closo-carborane	[31]
	NiTCP	ortho-closo-carborane	[34],[35]
	H2TCP	ortho-nido-carborane	[36],[37],[38],[39],[40]
	BOPP	closo-carborane	[6,41][42]
Phtalocyanin	ZnB_4Pc	closo-carborane	[43]
Chlorin	TPFC	ortho-nido-carborane	[44],[45]
	Fluorinated tetracarbachlorin	closo-carborane	[46]
Imaging agent	Gadolinium	ortho-closo-carborane<br>ortho-closo-carborane<br>ortho-closo-carborane<br>ortho-closo-carborane	[6],[36],[47],[48]

	Copper radioactive isotope (64Cu)	closo-carborane	[49]
		COSAN	[50]
		ortho-closo-carborane	[51]
	Iodine radioactive isotope	ortho-nido-carborane	[52],[53],[54],[55],[56],[57]
		para-closo-carborane	[58],[59],[60]
		COSAN	[61]
	Actinium radioactive isotope	ortho-nido-carborane	[55]
aminoacid	Phenylalnine	ortho-closo-carborane	[49]
Peptide	cRGD	ortho-closo-carborane	[62]
	PSMA	nido-carborane	[56]
		ortho-closo-carborane	[63]
	CPP (TAT)	ortho-closo-carborane	[64]
Antibodies	Mu-9<br>T84.66<br>T84.66<br>FAb′ of 107-1A4	ortho-nido-carborane	[52],[53],[54],[55]

Table S2. List of the drug delivery systems tested in in vivo BNCT.

Delivery System Category	Delivery System Components	Carborane Core	Ref.
Cyclodextrin	Cyclodextrin	(sulfonamide)-closo-carborane	[22]
Liposome	DSCP and cholesterol	nido-carborane	[65],[57],[66],[67]
	Cholesterol	nido-carborane	[68]
	Egg PC, DSPE-PEG	meta-closo-carborane	[69]
	Lecithin and cholesterol	nido-carborane	[70]
		ortho-closo-carborane	[71]
	PEG-DSPE, DSPC, cholesterol	ortho-closo-carborane	[49], [72]
	PEG	ortho-closo-carborane	[73]
		meta-closo-carborane	[74]
		nido-carborane	[75]
	DPPC, DPPG, cholesterol, PEG-2000	nido-carborane	[49]
	PEG and transferrin	nido-carborane	[57]
	PDNA, iRGD and cholesterol	ortho-closo-carborane	[23]
	Carborane-PEG	ortho-closo-carborane	[76]<br>[77]
Micelle	PEG	ortho-closo-carborane	[59],[60]
	MPEG-Galactose	ortho-closo-carborane	[78]
	TAT coated with hyaluronic acid	ortho-closo-carborane	[64]
	PLLA	ortho-closo-carborane	[79]
Nanoparticle	Magnetic Nanoparticle	ortho-closo-carborane	[80]
		meta-closo-carborane	[81]
	PLMB	ortho-closo-carborane	[73]
	Gold fluorescent nanoparticle (GNC)	nido-carborane	[82]
	PLGA / PLLGA	ortho-closo-carborane (pristine)	[83]
	Mesoporous silica	ortho-closo-carborane<br>ortho-closo-carborane (pristine)	[84]<br>[85]
	SWCNT	nido-carborane	[86]
	Gold Nanoparticle	COSAN	[87]<br>[88]
	Graphene-Oxide	COSAN	[89]
Covalent Organic	Porphyrine polymer	ortho-closo-carborane (pristine)	[51]

Frameworks (COF)	MOF	Para-closo-carborane	[90]
Nanogel	Pullulan	ortho-closo-carborane	[3]<br>[4]
LDL bionanoparticle	LDL	ortho-closo-carborane	[47],[48]
		nido-carborane	[91]

Table S3. Formulation used for the administration of the carborane-based drug in in vivo BNCT.

Solvent/ Formulant Class	Solvent/ Formulant Specie	Formulation		Carborane Core	Ref.
Aqueous	Water	CD	Porphyrin	ortho-nido-carborane	[44]
	PBS	CD	Porphyrin	ortho-closo-carborane	[33]
				ortho-nido-carborane	[40]
		DDS	Liposome	ortho-carborane	[71]
				meta-closo-carborane	[83]
				nido-closo-carborane	[65], [75]
			Nanoparticles	ortho-closo-carborane	[85]
Organic Solvent	DMF (traces)	CD	Targeting	ortho-nido-carborane	[52]
				ortho-closo-carborane	[64]
	EtOH	CD	Drug	nido-carborane	[43]
	DMSO	CD	Nucleoside	ortho-closo-carborane	[5],[7],[10],[11][12],[13][14]
				meta-nido-carborane	[15]
		DDS	Nanoparticles	ortho-closo-carborane	[80],[83]
				nido-carborane	[86]
	DMSO and peanut oil	CD	Drug	ortho-closo-carborane	[21]
	DMSO, PEG and H_2O	CD	Porphyrin	ortho-nido-carborane	[37]
	MeOH, DMSO, PEG, H_2O	CD	Porphyrin	ortho-nido-carborane	[38]
	DMSO and PBS	CD	Peptide	ortho-closo-carborane	[63]
	DMF and $CHCl_3$	DDS	Lactosome	ortho-closo-carborane	[79]
Formulant	CRM, PRG (propylene glycole) and NaCl	CD	Porphyrin 34	ortho-closo-carborane	[24],[25][26][27][31][34],[35]
				ortho-nido-carborane	[36]

Table S4. Classification of the in vivo BNCT studies based on the type of cancer investigated (cancer class), cell line used and its origin (human or murine).

Cancer Class	Cell line (origin)	Murine model	Formulation		Carborane core	Ref.
Brain	9LGS (murine)	BALB/c	CD	Porphyrin	ortho-closo-carborane	[25]
		Fischer rats	CD	Nucleoside	ortho-closo-carborane	[7]
	C6 (murine)	C57BL/6	CD	Porphyrin	ortho-closo-carborane	[46]
		Wistar rats	CD	Carbohydrate	ortho-closo-carborane	[1]
	F98 (murine)	Fischer rats	CD	Porphyrin	ortho-nido-carborane	[45],[40]
				Nucleoside	ortho-closo-carborane	[11],[13]
	GL261 (murine)	C57BL/6	DDS	Liposome	1-bromomethyl-o-carborane	[23]
			CD	Porphyrin	ortho-nido-carborane	[40]
	RG2 (murine)	Fischer rats	CD	Nucleoside	meta-nido-carborane	[15]
		NIH NCr-nu/nu	CD	Nucleoside	ortho-closo-carborane	[12]
	U373 (human)	BALB/c, C3H	CD	Porphyrin	ortho-closo-carborane	[31]
		BALB/c	CD	Porphyrin	ortho-closo-carborane	[25]
	U87 MG (human)	SCID	DDS	Liposome	ortho-closo-carborane	[72]
		BALB/c	CD	Drug	ortho-closo-carborane	[18]
		nu/nu	DDS	Vescicles	ortho-closo-carborane	[77]
		Fischer rats, SCID	CD	Porphyrin	ortho-closo-carborane	[25]
Breast	4T1 (murine)	BALB/c	DDS	Liposome	ortho-closo-carborane	[49]
				Micelle	ortho-closo-carborane	[79] [64]
				Nanoparticles	ortho-closo-carborane	[51]
	BCAP-37 (human)	BALB/c	DDS	Nanoparticles	ortho-closo-carborane	[80]
	BT-474 (human)	NOD	CD	Imaging Agent	COSAN	[50]
	EMT6 (murine)	C3H, BALB/c	CD	Porphyrin	ortho-closo-carborane	[31]
		BALB/c	CD	Porphyrin	ortho-closo-carborane	[30] [32] [24] [25] [29] [44]
			DDS	Nanoparticles	nido-carborane	[86]
				Liposome	nido-carborane	[35] [70] [65]
	Her2+ (human)	BALB/c, tEML4-ALK	DDS	Protein	ortho-closo-carborane	[48]
	KHJJ (murine)	BALB/c	CD	Porphyrin	ortho-closo-carborane	[35] [34] [33]
Colon	Colon-26 (murine)	BALB/c	CD	Carbohydrate	ortho-closo-carborane	[4]
			DDS	Micelle	ortho-closo-carborane	[59] [60]
				Liposome	ortho-nido-carborane	[57]
	CT26 (murine)	BALB/c	DDS	Liposome	nido-carborane	[75]
	MC38 (murine)	C57BL/6	DDS	COF	Para-carborane	[90]

	GW-39 (human)	nude mice xenografts	CD	Targeting	ortho-nido-carborane	[52]
	LS-174T (human)	nude mice xenografts	CD	Targeting	ortho-nido-carborane	[53] [54]
Connective Tissue	AB22 (murine)	BALB/c	DDS	Cyclodextrin	(sulfonamide)-Closo-carborane	[22]
	CMS5a (murine)	BALB/c	DDS	Nanogel	ortho-closo-carborane	[3]
	Exophytic (induced)	Syrian Hamster	DDS	Liposome	nido-carborane	[66] [66]
			CD	Porphyrin	ortho-closo-carborane	[28] [27]
	KHT (murine)	C3H	CD	Drug	ortho-closo-carborane	[21]
	HT1080	Nude mice (n.s.)	DDS	Nanoparticles	COSAN	[87]
	L929 (murine)	C3H	CD	Nucleoside	ortho-closo-carborane	[14]
		NIH nu/nu	CD	Nucleoside	ortho-closo-carborane	[13]
	SCCVII (murine)	C3H	CD	Nucleoside	ortho-closo-carborane	[62]
		BALB/c	CD	Porphyrin	ortho-closo-carborane	[29]
		BALB/c, C3H	CD	Porphyrin	ortho-closo-carborane	[31]
		C3H / H mice	CD	Drug	ortho-closo-carborane	[21]
Liver	AH109A (murine)	Donryu rats	CD	Drug	ortho-closo-carborane	[6]
	Hepa1-6 (murine)	C57BL/6	CD	Carbohydrate	ortho-closo-carborane	[2]
	H22 (murine)	BALB/c	DDS	Nanoparticles	ortho-closo-carborane	[85]
				Micelle	ortho-closo-carborane	[78]
Pancreas	PANC-1 (human)	ns	DDS	Nanoparticles	ortho-closo-carborane	[84]
Gastro-	MKN-45 (Human)	Nude mice Rj:NMR1	DDS	Nanoparticles	COSAN	[88]
Reproductive	22Rv1 (human)	nu/nu	CD	Peptide	ortho-closo-carborane	[62]
	9479 (human)	nude mice xenografts	CD	Nucleoside	ortho-closo-carborane	[8]
	LNCaP (human)	CD1	CD	Peptide	nido-carborane	[55]
		nude mice xenografts	CD	Nucleoside	ortho-closo-carborane	[8]
	not specified	SCID	CD	Nucleoside	ortho-closo-carborane	[16]
	HeLa (human)	nude mice xenografts	DDS	Nanoparticles	nido-carborane	[82]
	SKOV-3 (human)	CD1	CD	Imaging	para-closo-carborane	[58]
	U14 (murine)	Sprague–Dawley, KM	DDS	Nanoparticles	ortho-closo-carborane	[92]
		Sprague–Dawley, KM	DDS	Nanoparticles	ortho-closo-carborane	[73]
Skin	B16 (murine)	ddY	DDS	Nanoparticles	ortho-closo -carborane	[83]
				Liposome	meta-closo-carborane	[37]
					ortho-closo-carborane	[71]
		C57BL/6	DDS	COF	para-carborane	[90]
				Protein	ortho-closo-carborane	[47]
			CD	Porphyrin	ortho-nido-carborane	[43] [38]

						[46]
				Drug	nido-carborane	[20]
				Phtalocyanin	closo-carborane	[43]
				Imaging	ortho-closo-carborane	[69]
	Harding-Passey	BALB/c	CD	Nucleoside	ortho-nido-carborane	[17]
			DDS	Liposome	nido-carborane	[91]
	MM-138 (human)	nude mice xenografts	CD	Porphyrin	n.s.	[36]
Thyroid	ARO (hu-man)	NIH	CD	Porphyrin	closo-carborane	[41]
	UTC (hu-man)	NIH	CD	Porphyrin	closo-carborane	[42]
Biodistri-bution / toxicity	non-tu-moral	n.s.	DDS	Graphene Oxide	COSAN	[89]
				Nanoparticles	meta-closo-carborane	[81]
				Liposome	ortho-closo-carborane	[76]
				Imaging	COSAN	[61]
			CD	Nucleoside	ortho-closo-carborane	[5]
		Fische rats	CD	Porphyrin	ortho-closo-carborane	[26]
		BALB/c	CD	Pristine	COSAN	[93]
				Drug	closo-carborane	[19]
				Targeting (mAb)	ortho-nido-carborane	[55]
			DDS	Liposome	nido-carborane	[68]
		C57BL/6	CD	Nucleoside	ortho-closo-carborane	[10]

Table S5. In vivo BNCT carborane studies employing prompt gamma-ray neutron activation analysis (PGAA) for the analysis of boron content.

<table>
<tr><th>Analytical method</th><th>Carborane structure</th><th colspan="2">Formulation</th><th><u>Tumor Model</u>
Cell line

Animal model</th><th>ref</th></tr>
<tr><td rowspan="8">PGAA</td><td rowspan="3">ortho-carborane</td><td rowspan="3">CD</td><td rowspan="2">Porphyrin</td><td>U87, U373 and 9LGS (Brain);
EMT-6 (Breast)

Fisher rats,
SCID mice</td><td>[25]</td></tr>
<tr><td>EMT-6 (Breast)

BALB/c mice</td><td>[32]
[24]</td></tr>
<tr><td>Nucleoside</td><td>Non-tumoral

C57BL/6 mice</td><td>[10]</td></tr>
<tr><td rowspan="4">nido-carborane</td><td rowspan="4">CD</td><td rowspan="2">Porphyrin</td><td>KHJJ (Breast),
EMT-6 (Breast)

BALB/c mice</td><td>[35]</td></tr>
<tr><td>KHJJ (Breast)
----
n.s.</td><td>[33]</td></tr>
<tr><td>Nucleoside</td><td>Harding-Passey (Skin)

BALB/c mice</td><td>[17]</td></tr>
<tr><td>Drug</td><td>B16 (Skin)

C57BL/6 mice</td><td>[20]</td></tr>
</table>

Table S6. In vivo BNCT carborane studies employing gamma-ray emission analysis for the detection of radionuclides-labelled carborane-conjugates.

**Analytical method**	**Carborane structure**	**Formulation**		Tumor Model<br>*Cell line*<br>---------<br>*Animal model*	**ref**
I-125 gamma counter	ortho/nido carborane	CD	Antibody	GW-39 (Colon)<br>---------<br>Nude mice (n.s.)	[52]
	nido-carborane	CD	Antibody	LS174T (Colon)<br>---------<br>Nude mice (n.s.)	[53]
					[54]
			Peptide	LNCaP (Prostate)<br>---------<br>CD1 mice	[55]
		DDS	Liposome	Colon-26 (Colon)<br>---------<br>BALB/c mice	[57]
	ortho-carborane	DDS	Micelle	Colon-26 (Colon)<br>---------<br>BALB/c mice	[59], [60]
	COSAN	DDS	PEG	Non tumoral<br>---------<br>n.s.	[61]
I-124 gamma counter	COSAN		Graphene Oxide	HT1080 (Conn. Tissue)<br>---------<br>Nude mice (n.s.)	[88]
At-211 and I-125 gamma counter	nido-carborane	CD	Antibody	Non-tumoral<br>---------<br>BALB/c mice	[55]
Cu-64 gamma counter	ortho-carborane	DDS	Liposome	4T1 (Breast)<br>---------<br>BALB/c mice	[51]
Ga-68 gamma counter	ortho-carborane	CD	Peptide	22Rv1 (Prostate)<br>---------<br>Athymic mice	[62]

Table S7. In vivo BNCT carborane studies employing plasma-based techniques for the (*ex vivo*) elemental analysis of boron. Direct current plasma (DCP) and inductively coupled plasma (ICP) were coupled to either optical emission spectroscopy (OES) or mass spectrometry (MS).

Analytical method	Carborane structure	Formulation		Tumor Model<br>*Cell line*<br>---------<br>*Animal model*	Ref.
DCP-OES	ortho-carborane	CD	Porphyrin	KHJJ (Breast)<br>---------<br>BALB/c mice	[34]
				U87, U373 and 9LGS (Brain)<br>EMT-6 (Breast)<br>---------<br>Fisher rats<br>SCID mice	[25]
				EMT-6 (Breast)<br>---------<br>BALB/c mice	[24]
				EMT-6 (Breast)<br>---------<br>BALB/c mice	[32]
				EMT-6 (Breast)<br>---------<br>BALB/c mice	[30]
				EMT-6 (Breast) and SCCVII (Squamous cells)<br>---------<br>BALB/c mice<br>C3H mice	[29]
				U373 (Brain), EMT-6 (Breast), SCCVII (Squamous cells)<br>---------<br>BALB/c mice<br>C3H mice	[31]
				Non-tumoral<br>---------<br>Fisher rats	[26]
			Nucleosides	F98 (Brain)<br>L929 (Conn. tissue)<br>---------<br>Fischer rats<br>C3H mice	[13]
				F98 (Brain)<br>---------<br>Rats (n.s.)	[11]
				RG2 (Brain)<br>---------<br>NIH mice	[12]
				9L (Brain)<br>---------<br>Fischer rats	[7]
	nido-carborane	CD	Porphyrin	GL261(Brain) and F98 (Brain)<br>---------<br>C57BL/6 mice	[40]
				EMT-6 (Breast)<br>---------<br>BALB/c mice	[44]

				KHJJ (Breast)<br>EMT-6 (Breast)<br>---------<br>BALB/c mice	[35]
			Nucleosides	L929 (Conn. tissue)<br>---------<br>C3H mice	[14]
ICP-OES	ortho-carborane	CD	Carbohydrate	C6 (Brain)<br>---------<br>Wistar rats	[1]
				Colon-26 (Colon)<br>---------<br>BALB/c mice	[4]
			Porphyrin	Exophytic-induced<br>(Oral carcinoma)<br>---------<br>Syrian Hamster	[27]
				MM-138 (Skin)<br>---------<br>Nude mice (n.s.)	[36]
				Exophytic-induced<br>(Oral carcinoma)<br>---------<br>Syrian Hamster	[28]
				UTC (Thyroid)<br>---------<br>NIH mice	[41,42]
			Chlorine	F98 (Brain)<br>---------<br>Fischer rats	[45]
			Nucleosides	RG2 (Brain)<br>---------<br>Fischer rats	[15]
				SCCVII (Squamous cells)<br>---------<br>CH3 mice	[62]
				AH109A (Liver)<br>---------<br>Donryu rats	[6]
			Peptide	22Rv1 (Prostate)<br>---------<br>Athymic mice	[62]
		DDS	Liposome	B16 (Skin)<br>---------<br>DdY mice	[71]
				U87 (Brain)<br>---------<br>SCID mice	[72]
				4T1 (Breast)<br>---------<br>BALB/c mice	[49]
				Non-tumoral<br>---------<br>n.s.	[76]
			Micelle	4T1 (Breast)<br>---------<br>BALB/c mice	[64]
				4T1 (Breast)<br>---------	[79]

				BALB/c mice	
			Nanoparticles	B16 (Skin)<br>---------<br>DdY mice	[83]
				BCAP-37 (Breast)<br>---------<br>BALB/c mice	[80]
			COP	4T1 (Breast)<br>---------<br>BALB/c mice	[51]
	meta-carborane	DDS	Liposome	B16 (Skin)<br>---------<br>DdY mice	[69]
	nido-carborane	DDS	Nanoparticle	EMT-6 (Breast)<br>---------<br>BALB/c mice	[86]
		DDS	Liposome	EMT-6 (Breast)<br>---------<br>BALB/c mice	[70]
		DDS		Non-tumoral<br>---------<br>BALB/c mice	[68]
	COSAN	/	Pristine	Non-tumoral<br>---------<br>BALB/c mice	[19]
ICP-MS	ortho-carborane	DDS	LDL	Her2+ (Breast)<br>---------<br>BALB/c, transgenic EML4-ALK	[48]
			Nanoparticles	U14 (Cervix)<br>---------<br>Sprague− Dawley rats, KM mice	[73]
				PANC-1 (Pancreas)<br>---------<br>n.s.	[84]
				Hepa1-6 (Liver)<br>---------<br>C57BL/6	[2]
				H22 (Liver)<br>---------<br>BALB/c mice	[85]
			Cyclodextrins	AB22 (Mesothelium)<br>---------<br>BALB/c mice	[22]
			Micelle	Colon-26 (Colon)<br>---------<br>BALB/c mice	[59]
				Colon-26 (Colon)<br>---------<br>BALB/c mice	[60]
				H22 (Liver)<br>---------<br>BALB/c mice	[78]
			Liposome	U14 (Cervix)<br>---------<br>KM mice	[92]
	nido-carborane	DDS	Liposome	EMT-6 (Breast)<br>---------	[65]

				BALB/c mice	
				Harding-Passey (skin)<br>---------<br>BALB/c mice	[91]
				Exophytic-induced<br>(Oral carcinoma)<br>---------<br>Syrian Hamster	[66]
				Exophytic-induced<br>(Oral carcinoma)<br>---------<br>Syrian Hamster	[66]
				CT26 (Colorectal)<br>---------<br>BALB/c mice	[75]
	COSAN	DDS	Graphene Oxide	HT1080 (Conn. Tissue)<br>---------<br>Nude mice (n.s.)	[88]

Table S8. In vivo BNCT carborane studies employing fluorescence-based techniques for the detection of carborane-conjugates.

Fluorescence Analysis	Fluorophore	Carborane structure	Formulation		Tumor Model<br>*Cell line*<br>---------<br>*Animal model*	Ref.
Ex vivo	Chlorin cluster	ortho-carborane	CD	Chlorine	B16 (Skin)<br>C6 (Brain)<br>---------<br>C57BL6 or Balb/c mice	[46]
				Chlorine	F98 (Brain)<br>---------<br>Fischer rats	[45]
	NIR (PN50, PN90, PN150)	ortho-carborane	DDS	Liposome	U14 (Cervix)<br>---------<br>KM mice	[92]
	Phalocyanine cluster	ortho-carborane	CD	Phalocyanine	B16 (Skin)<br>---------<br>C57BL/6 mice	[43]
	Porphyrin cluster	nido-carborane	CD	Porphyrin	B16 (Skin)<br>---------<br>C57BL/6 mice	[37]
						[38]
						[39]
	VivoTrack 680	nido-carborane	DDS	Liposome	CT26 (Colorectal)<br>---------<br>BALB/c mice	[75]
In vivo	Au-NPs	nido-carborane	DDS	Nanoparticle	HeLa (Cervix)<br>---------<br>Nude mice (n.s.)	[82]
	DiR,Cy5.5	ortho-carborane	DDS	Nanoparticle	Hepa1-6 (Liver)<br>---------<br>C57BL/6	[2]
	Doxorubicin	ortho-carborane	DDS	Vescicles	U87 (Brain)<br>---------<br>nu/nu mice	[77]
				Nanoparticle	U14 (Cervix)<br>---------<br>Sprague– Dawley rats, KM mice	[73]
					PANC-1 (Pancreas)<br>---------<br>n.s.	[84]
	Indocyanine Green	ortho-carborane	DDS	Micelle	4T1 (Breast)<br>---------<br>BALB/c mice	[79]
	Luciferin	meta-carborane	CD	Drug	U87 (Brain)<br>---------<br>BALB/c mice	[88]
		ortho-carborane	DDS	Liposome	Non-tumoral<br>---------<br>n.s.	[76]

	Rhodamin B			Micelle	H22 (Liver)<br>---------<br>BALB/c mice	[78]
				Nanogel	CMS5a<br>(Conn. Tissue)<br>---------<br>BALB/c mice	[3]

Table S9. In vivo BNCT studies employing tomography-based techniques for the imaging of the carborane drugs.

Analytical method	Tracer	Carborane structure	Formulation		Tumor Model<br>*Cell line*<br>---------<br>*Animal model*	Ref.
PET-CT	^{64}Cu	ortho-carborane	DDS	Liposome	4T1 (Breast)<br>---------<br>BALB/c mice	[49],[51]
		COSAN	DDS	Nanoparticle	BT-474 (Breast)<br>---------<br>NOD/SCID mice	[50]
					MKN-45 (Gastrointestinal)<br>---------<br>Nude mice<br>Rj:NMRI-Fox1nu/nu	[88]
	^{124}I	COSAN	DDS	PEG	Non tumoral<br>---------<br>n.s.	[61]
	^{89}Zr	Para-carborane	DDS	COF	B16F10 (Skin<br>MC38 (Colorectal)<br>---------<br>C57BL6 mice	[90]
PET	^{124}I	COSAN	DDS	Nanoparticle	HT1080 (Conn. Tissue)<br>---------<br>Nude mice (n.s.)	[87]
				Graphene Oxide	Non-tumoral<br>---------<br>Mice (wild type)	[89]
	^{68}Ga	ortho-carborane	CD	Lipids	22Rv1 (Prostate)<br>---------<br>Athymic mice	[62]
SPECT-CT	^{123}I	nido-carborane	CD	Drug	SKOV-3 (Ovary)<br>---------<br>CD1 mice	[58]

Table S10. In vivo BNCT studies employing magnetic resonance imaging (MRI) for the imaging of the carborane drugs

Analytical method	Tracer	Carborane structure	Formulation (Contrast agent)		Tumor Model<br>*Cell line*<br>---------<br>*Animal model*	Ref.
Magnetic resonance imaging (MRI)	Gd	ortho-carborane	CD	Nucleoside	AH109A (Liver)<br>---------<br>Donryu rats	[6]
			DDS	B-CD / LDL	B16 (Skin)<br>---------<br>C57BL/6 mice	[47]
					Her2+ (Breast)<br>---------<br>BALB/c, transgenic EML4-ALK	[48]
	None<br>(^{11}B detection)	nido-carborane	CD	Drug	SCCVII (Squamous cells)<br>KHT (Conn. Tissue)<br>---------<br>C3H mice	[21]

References

1. Tietze, L.F.; Bothe, U.; Griesbach, U.; Nakaichi, M.; Hasegawa, T.; Nakamura, H.; Yamamoto, Y. Ortho-Carboranyl Glycosides for the Treatment of Cancer by Boron Neutron Capture Therapy. *Bioorg & Med Chem* **2001**, *9*, 1747-1752, doi.org/10.1016/S0968-0896(01)00061-X.
2. Yang, Q.; Dai, Q.; Bao, X.; Zhou, Y.; Lu, Y.; Zhong, H.; Wu, L.; Guo, Y.; Liu, L.; Tan, X.; et al. Evaluation of a Tumor-Targeting Oligosaccharide Nanosystem in BNCT on an Orthotopic Hepatocellular Carcinoma Model. *Mol Pharm* **2023**, *20*, 1025–1038, doi:10.1021/acs.molpharmaceut.2c00771.
3. Kawasaki, R.; Sasaki, Y.; Akiyoshi, K. Intracellular Delivery and Passive Tumor Targeting of a Self-Assembled Nanogel Containing Carborane Clusters for Boron Neutron Capture Therapy. *Biochem Biophys Res Commun* **2017**, *483*, 147–152, doi:10.1016/j.bbrc.2016.12.176.
4. Kawasaki, R.; Hirano, H.; Yamana, K.; Isozaki, H.; Kawamura, S.; Sanada, Y.; Bando, K.; Tabata, A.; Yoshikawa, K.; Azuma, H.; et al. Carborane Bearing Pullulan Nanogel-Boron Oxide Nanoparticle Hybrid for Boron Neutron Capture Therapy. *Nanomedicine* **2023**, *49*, 102659, doi:10.1016/j.nano.2023.102659.
5. Jarugula, V.R.; Schinazi, R.F.; Fulcrand, G.; El Kattan, Y.; Liotaii, D.C.; Douglas Boudinot, F. Pharmacokinetics of 5-Carboranyl-2′-deoxyuridine in Rats. *J Pharm Sci,* **1994**, 83, 1597-1599. ~, Y.; Liotaii, D.C.; Douglas Boudinot, F. *P Harmaco Ki Net Ics of 5-Carboranyl-2′-Deoxyurid i Ne in Rats;*
6. Nakamura, H.; Fukuda, H.; Girald, F.; Kobayashi, T.; Hiratsuka, J.; Akaizawa, T.; Nemoto, H.; Cai, J.; Yoshida, K.; Yamamoto, Y. In Vivo Evaluation of Carborane Gadolinium-DTPA Complex as an MR Imaging Boron Carrier. *Chem Pharm Bull (Tokyo)* **2000**, *48*, 1034–1038, doi:10.1248/cpb.48.1034.
7. Schinazi, R.F.; Hurwitz, S.J.; Liberman, I.; Juodawlkis, A.S.; Tharnish, P.; Shi, J.; Liotta, D.C.; Coderre, J.A.; Olson, J. Treatment of Isografted 9L Rat Brain Tumors with β-5-o-Carboranyl-2′- Deoxyuridine Neutron Capture Therapy. *Clinical Cancer Research* **2000**, *6*, 725–730.
8. Schinazi, R.F.; Hurwitz, S.J.; Liberman, I.; Glazkova, Y.; Mourier, N.S.; Olson, J.; Keane, T. Tissue Disposition of 5-o-Carboranyluracil - A Novel Agent for the Boron Neutron Capture Therapy of Prostate Cancer. *Nucleosides Nucleotides Nucleic Acids* **2004**, *23*, 291–306, doi:10.1081/NCN-120027836.
9. Barth, R.F.; Yang, W.; Al-Madhoun, A.S.; Johnsamuel, J.; Byun, Y.; Chandra, S.; Smith, D.R.; Tjarks, W.; Eriksson, S. Boron-Containing Nucleosides as Potential Delivery Agents for Neutron Capture Therapy of Brain Tumors. *Cancer Res* **2004**, *64*, 6287–6295, doi:10.1158/0008-5472.CAN-04-0437.
10. Binello, E.; Mitchell, R.N.; Harling, O.K. T Cell Uptake for the Use of Boron Neutron Capture as an Immunologic Research Tool. *App Rad Isot,* **2004**, 61, 959–962, doi: 10.1016/j.apradiso.2004.05.019.

11. Byun, Y.; Thirumamagal, B.T.S.; Yang, W.; Eriksson, S.; Barth, R.F.; Tjarks, W. Preparation and Biological Evaluation of 10B-Enriched 3-[5-{2-(2,3-Dihydroxyprop-1-Yl)-o-Carboran-1-Yl}pentan-1-Yl]Thymidine (N5-2OH), a New Boron Delivery Agent for Boron Neutron Capture Therapy of Brain Tumors. *J Med Chem* **2006**, *49*, 5513–5523, doi:10.1021/jm060413w.
12. Barth, R.F.; Yang, W.; Wu, G.; Swindall, M.; Byun, Y.; Narayanasamy, S.; Tjarks, W.; Tordoff, K.; Moeschberger, M.L.; Eriksson, S.; et al. Thymidine Kinase 1 as a Molecular Target for Boron Neutron Capture Therapy of Brain Tumors. *Proc Natl Acad Sci U S A* **2008**, *105*, 17493–17497, doi:10.1073/pnas.0809569105.
13. Barth, R.F.; Yang, W.; Al-Madhoun, A.S.; Johnsamuel, J.; Byun, Y.; Chandra, S.; Smith, D.R.; Tjarks, W.; Eriksson, S. Boron-Containing Nucleosides as Potential Delivery Agents for Neutron Capture Therapy of Brain Tumors. *Canc Res*, **2004**, *64*, 6287-6295, DOI: 10.1158/0008-5472.CAN-04-0437 .
14. Byun, Y.; Yan, J.; Al-Madhoun, A.S.; Johnsamuel, J.; Yang, W.; Barth, R.F.; Eriksson, S.; Tjarks, W. Synthesis and Biological Evaluation of Neutral and Zwitterionic 3-Carboranyl Thymidine Analogues for Boron Neutron Capture Therapy. *J Med Chem* **2005**, *48*, 1188–1198, doi:10.1021/jm0491896.
15. Agarwal, H.K.; Khalil, A.; Ishita, K.; Yang, W.; Nakkula, R.J.; Wu, L.C.; Ali, T.; Tiwari, R.; Byun, Y.; Barth, R.F.; et al. Synthesis and Evaluation of Thymidine Kinase 1-Targeting Carboranyl Pyrimidine Nucleoside Analogs for Boron Neutron Capture Therapy of Cancer. *Eur J Med Chem* **2015**, *100*, 197–209, doi:10.1016/j.ejmech.2015.05.042.
16. Goudgaon, N.M.; El-Kattan, Y.A.; Xia, X.; McAtee, J.; Soria, J.; Wey, S.J.; Liotta, D.C.; Schinazi, R.F. A General Synthetic Method of 5-Carboranyluracil Nucleosides with Potential Antiviral Activity and Use in Neutron Capture Therapy. *Nucleosides Nucleotides* **1997**, *16*, 2133–2150, doi:10.1080/07328319708002564.
17. Reynolds, R.C.; Campbell, S.R.; Fairchild, R.G.; Kisliuk, R.L.; Micca, P.L.; Queener, S.F.; Riordan, J.M.; Sedwick, W.D.; Waud, W.R.; Leung, A.K.W.; et al. Novel Boron-Containing, Nonclassical Antifolates: Synthesis and Preliminary Biological and Structural Evaluation. *J Med Chem* **2007**, *50*, 3283–3289, doi:10.1021/jm0701977.
18. Alamón, C.; Dávila, B.; García, M.F.; Sánchez, C.; Kovacs, M.; Trias, E.; Barbeito, L.; Gabay, M.; Zeineh, N.; Gavish, M.; et al. Sunitinib-Containing Carborane Pharmacophore with the Ability to Inhibit Tyrosine Kinases Receptors FLT3, KIT and PDGFR-β, Exhibits Powerful in Vivo Anti-Glioblastoma Activity. *Cancers (Basel)* **2020**, *12*, 1–21, doi:10.3390/cancers12113423.
19. Couto, M.; Alamón, C.; Sánchez, C.; Dávila, B.; Fernández, M.; Lecot, N.; Cabral, P.; Teixidor, F.; Viñas, C.; Cerecetto, H. Carboranylanilinoquinazoline EGFR-Inhibitors: Toward “lead-to-Candidate” Stage in the Drug-Development Pipeline. *Future Med Chem* **2019**, *11*, 2273–2285, doi:10.4155/fmc-2019-0060.
20. Morris, J.H.; Peters, G.S.; Koldaeva, E.; Spryshkova, R.; Borisov, G. Synthesis and Characterization of 7-(CH3)3N-4- {2,4-(NO2)2C6H3S}-nido-7-CB10H11 and Its Biodistribution in C57B16 Mice Bearing B16 Melanoma. *Appl Organomet Chem* **1995**, *9*, 323–325, doi:10.1002/aoc.590090405.
21. Wood, P.J.; Scobie, M.; Threadgill, M.D. Uptake and Retention of Nitroim Idazole-Carboranes Designed for Boron Neutron Capture Therapy in Experim Ental m Urine Tum Ours: Detection by 11 B m Agnetic Resonance Spectroscopy. *Int J Radiat Biol*, **1996**, *70*, 587-592, doi: 10.1080/095530096144798.
22. Alberti, D.; Michelotti, A.; Lanfranco, A.; Protti, N.; Altieri, S.; Deagostino, A.; Geninatti Crich, S. In Vitro and in Vivo BNCT Investigations Using a Carborane Containing Sulfonamide Targeting CAIX Epitopes on Malignant Pleural Mesothelioma and Breast Cancer Cells. *Sci Rep* **2020**, *10*, doi:10.1038/s41598-020-76370-1.
23. Chen, J.; Dai, Q.; Yang, Q.Y.; Bao, X.; Zhou, Y.; Zhong, H.; Wu, L.; Wang, T.; Zhang, Z.; Lu, Y.; et al. Therapeutic Nucleus-Access BNCT Drug Combined CD47-Targeting Gene Editing in Glioblastoma. *J Nanobiotechnology* **2022**, *20*, 1–18, doi:10.1186/s12951-022-01304-0.
24. Miura, M.; Morris, G.M.; Micca, P.L.; Lombardo, D.T.; Youngs, K.M.; Kalef-Ezra, J.A.; Hoch, D.A.; Slatkin, D.N.; Ma, R.; Coderre, J.A. Boron Neutron Capture Therapy of a Murine Mammary Carcinoma Using a Lipophilic Carboranyltetraphenylporphyrin. *Radiat Res* **2001**, *155*, 603–610, doi:10.1667/0033-7587(2001)155[0603:BNCTOA]2.0.CO;2.
25. Miura, M.; Joel, D.D.; Smilowitz, H.M.; Nawrocky, M.M.; Micca, P.L.; Hoch, D.A.; Coderre, J.A.; Slatkin, D.N. Biodistribution of Copper Carboranyltetraphenylporphyrins in Rodents Bearing an Isogeneic or Human Neoplasm. *J Neurooncol* **2001**, *52*, 111–117, doi:10.1023/A:1010622319892.
26. Morris, G.M.; Coderre, J.A.; Hopewell, J.W.; Micca, P.L.; Nawrocky, M.; Miura, M. Porphyrin-Mediated Boron Neutron Capture Therapy: Evaluation of the Reactions of Skin and Central Nervous System. *Int J Radiat Biol* **2003**, *79*, 149–158, doi:10.1080/0955300031000073392.

27. Kreimann, E.L.; Miura, M.; Itoiz, M.E.; Heber, E.; Garavaglia, R.N.; Batistoni, D.; Jiménez Rebagliati, R.; Roberti, M.J.; Micca, P.L.; Coderre, J.A.; et al. Biodistribution of a Carborane-Containing Porphyrin as a Targeting Agent for Boron Neutron Capture Therapy of Oral Cancer in the Hamster Cheek Pouch. *Arch Oral Biol* **2003**, *48*, 223–232, doi:10.1016/S0003-9969(02)00210-8.
28. Stoliar, P.; Kreiner, A.J.; Debray, M.E.; Caraballo, M.E.; Valda, A.A.; Davidson, J.; Davidson, M.; Kesque, J.M.; Somacal, H.; DiPaolo, H.; et al. Microdistributions of Prospective BNCT-Compound CuTCPH in Tissue Sections with a Heavy Ion Microbeam. *Applied Radiation and Isotopes,* **2004**, *61,* 771–774, doi.org/10.1016/j.apradiso.2004.05.062
29. Miura, M.; Morris, G.M.; Micca, P.L.; Nawrocky, M.M.; Makar, M.S.; Cook, S.P.; Slatkin, D.N. Synthesis of Copper Octabromotetracarboranylphenylporphyrin for Boron Neutron Capture Therapy and Its Toxicity and Biodistribution in Tumour-Bearing Mice. *British Journal of Radiology* **2004**, *77*, 573–580, doi:10.1259/bjr/71404908.
30. Miura, M.; Morris, G.M.; Hopewell, J.W.; Micca, P.L.; Makar, M.S.; Nawrocky, M.M.; Renner, M.W. Enhancement of the Radiation Response of EMT-6 Tumours by a Copper Octabromotetracarboranylphenylporphyrin. *British Journal of Radiology* **2012**, *85*, 443–450, doi:10.1259/bjr/87260973.
31. Smilowitz, H.M.; Slatkin, D.N.; Micca, P.L.; Miura, M. Microlocalization of Lipophilic Porphyrins: Non-Toxic Enhancers of Boron Neutron-Capture Therapy. *Int J Radiat Biol* **2013**, *89*, 611–617, doi:10.3109/09553002.2013.782446.
32. Wu, H.; Micca, P.L.; Makar, M.S.; Miura, M. Total Syntheses of Three Copper (II) Tetracarboranylphenylporphyrins Containing 40 or 80 Boron Atoms and Their Biological Properties in EMT-6 Tumor-Bearing Mice. *Bioorg Med Chem* **2006**, *14*, 5083–5092, doi:10.1016/j.bmc.2006.04.010.
33. Miura, M.; Micca, P.L.; Heinrichs, J.C.; Gabel, D.; Fairchild, R.G.; Slatkin, D.N. Biodistribution and Toxicity of 2,4-Divinyl-Nido-o-Carboranyldeuteroporphyrin IX in Mice. *Biochem Pharmacol* **1992**, *43*, 467–476, doi:10.1016/0006-2952(92)90565-Z.
34. Miura, M.; Micca, P.L.; Donaldson, J.A.; Heinrichs, J.C.; Shelnutt, J.A.; Finkel, G.C.; Slatkin, D.N. Synthesis, Tissue Uptake, and Toxicity of a Nickel Tetracarboranylphenylporphyrin. *Cancer Neutron Capture Therapy* **1996**, *119*, 137–141, doi:10.1007/978-1-4757-9567-7_18.
35. Miura, M.; Micca, P.L.; Fisher, C.D.; Gordon, C.R.; Heinrichs, J.C.; Slatkin, D.N. Evaluation of Carborane-Containing Porphyrins as Tumour Targeting Agents for Boron Neutron Capture Therapy. *British Journal of Radiology* **1998**, *71*, 773–781, doi:10.1259/bjr.71.847.9771389.
36. Shahbazi-Gahrouei, D.; Williams, M.; Rizvi, S.; Allen, B.J. In Vivo Studies of Gd-DTPA-Monoclonal Antibody and Gd-Porphyrins: Potential Magnetic Resonance Imaging Contrast Agents for Melanoma. *Journal of Magnetic Resonance Imaging* **2001**, *14*, 169–174, doi:10.1002/jmri.1168.
37. Fabris, C.; Vicente, M.G.H.; Hao, E.; Friso, E.; Borsetto, L.; Jori, G.; Miotto, G.; Colautti, P.; Moro, D.; Esposito, J.; et al. Tumour-Localizing and -Photosensitising Properties of Meso-Tetra(4-Nido-Carboranylphenyl)Porphyrin (H2TCP). *J Photochem Photobiol B* **2007**, *89*, 131–138, doi:10.1016/j.jphotobiol.2007.09.012.
38. Soncin, M.; Friso, E.; Jori, G.; Hao, E.; Vicente, M.G.H.; Miotto, G.; Colautti, P.; Moro, D.; Esposito, J.; Rosi, G.; et al. Tumor-Localizing and Radiosensitizing Properties of Meso-Tetra(4-Nido- Carboranylphenyl) Porphyrin (H2TCP). *J Porphyrins and Phatlocyanines*, **2008**, *12*, 866–873.
39. Jori, G.; Soncin, M.; Friso, E.; Vicente, M.G.H.; Hao, E.; Miotto, G.; Colautti, P.; Moro, D.; Esposito, J.; Rosi, G.; et al. A Novel Boronated-Porphyrin as a Radio-Sensitizing Agent for Boron Neutron Capture Therapy of Tumours: In Vitro and in Vivo Studies. *Applied Radiation and Isotopes* **2009**, *67*, doi:10.1016/j.apradiso.2009.03.071.
40. Kawabata, S.; Yang, W.; Barth, R.F.; Wu, G.; Huo, T.; Binns, P.J.; Riley, K.J.; Ongayi, O.; Gottumukkala, V.; Vicente, M.G.H. Convection Enhanced Delivery of Carboranylporphyrins for Neutron Capture Therapy of Brain Tumors. *J Neurooncol* **2011**, *103*, 175–185, doi:10.1007/s11060-010-0376-5.
41. Viaggi, M.; Dagrosa, M.A.; Longhino, J.; Blaumann, H.; Calzetta, O.; Kahl, S.B.; Juvenal, G.J.; Pisarev, M.A. Boron Neutron Capture Therapy for Undifferentiated Thyroid Carcinoma: Preliminary Results with the Combined Use of BPA and BOPP. *Applied Radiation and Isotopes,* **2004**, *61,* 905–909, doi: 10.1016/j.apradiso.2004.05.005.

42. Dagrosa, M.A.; Viaggi, M.; Rebagliati, R.J.; Batistoni, D.; Kahl, S.B.; Juvenal, G.J.; Pisarev, M.A. Biodistribution of Boron Compounds in an Animal Model of Human Undifferentiated Thyroid Cancer for Boron Neutron Capture Therapy. *Mol Pharm* **2005**, *2*, 151–156, doi:10.1021/mp049894a.
43. Friso, E.; Roncucci, G.; Dei, D.; Soncin, M.; Fabris, C.; Chiti, G.; Colautti, P.; Esposito, J.; De Nardo, L.; Riccardo Rossi, C.; et al. A Novel 10B-Enriched Carboranyl-Containing Phthalocyanine as a Radio- and Photo-Sensitising Agent for Boron Neutron Capture Therapy and Photodynamic Therapy of Tumours: In Vitro and in Vivo Studies. *Photochemical and Photobiological Sciences* **2006**, *5*, 39–50, doi:10.1039/b506364g.
44. Vicente, M.G.H.; Wickramasinghe, A.; Nurco, D.J.; Wang, H.J.H.; Nawrocky, M.M.; Makar, M.S.; Miura, M. Synthesis, Toxicity and Biodistribution of Two 5,15-Di[3,5-(Nido-Carboranylmethyl)Phenyl]Porphyrins in EMT-6 Tumor Bearing Mice. *Bioorg Med Chem* **2003**, *11*, 3101–3108, doi:10.1016/S0968-0896(03)00240-2.
45. Hiramatsu, R.; Kawabata, S.; Tanaka, H.; Sakurai, Y.; Suzuki, M.; Ono, K.; Miyatake, S.I.; Kuroiwa, T.; Hao, E.; Vicente, M.G.H. Tetrakis(p-Carboranylthio-Tetrafluorophenyl)Chlorin (TPFC): Application for Photodynamic Therapy and Boron Neutron Capture Therapy. *J Pharm Sci* **2015**, *104*, 962–970, doi:10.1002/jps.24317.
46. Ol'shevskaya, V.A.; Zaitsev, A. V.; Petrova, A.S.; Arkhipova, A.Y.; Moisenovich, M.M.; Kostyukov, A.A.; Egorov, A.E.; Koroleva, O.A.; Golovina, G. V.; Volodina, Y.L.; et al. The Synthetic Fluorinated Tetracarboranylchlorin as a Versatile Antitumor Photoradiosensitizer. *Dyes and Pigments* **2021**, *186*, 108993, doi:10.1016/j.dyepig.2020.108993.
47. Geninatti-Crich, S.; Alberti, D.; Szabo, I.; Deagostino, A.; Toppino, A.; Barge, A.; Ballarini, F.; Bortolussi, S.; Bruschi, P.; Protti, N.; et al. MRI-Guided Neutron Capture Therapy by Use of a Dual Gadolinium/Boron Agent Targeted at Tumour Cells through Upregulated Low-Density Lipoprotein Transporters. *Chemistry - A European Journal* **2011**, *17*, 8479–8486, doi:10.1002/chem.201003741.
48. Alberti, D.; Protti, N.; Toppino, A.; Deagostino, A.; Lanzardo, S.; Bortolussi, S.; Altieri, S.; Voena, C.; Chiarle, R.; Geninatti Crich, S.; et al. A Theranostic Approach Based on the Use of a Dual Boron/Gd Agent to Improve the Efficacy of Boron Neutron Capture Therapy in the Lung Cancer Treatment. *Nanomedicine* **2015**, *11*, 741–750, doi:10.1016/j.nano.2014.12.004.
49. Li, J.; Sun, Q.; Lu, C.; Xiao, H.; Guo, Z.; Duan, D.; Zhang, Z.; Liu, T.; Liu, Z. Boron Encapsulated in a Liposome Can Be Used for Combinational Neutron Capture Therapy. *Nat Commun* **2022**, *13*, doi:10.1038/s41467-022-29780-w.
50. Feiner, I.V.J.; Pulagam, K.R.; Gómez-Vallejo, V.; Zamacola, K.; Baz, Z.; Caffarel, M.M.; Lawrie, C.H.; Ruiz-de-Angulo, A.; Carril, M.; Llop, J. Therapeutic Pretargeting with Gold Nanoparticles as Drug Candidates for Boron Neutron Capture Therapy. *Particle and Particle Systems Characterization* **2020**, *37*, doi:10.1002/ppsc.202000200.
51. Shi, Y.; Fu, Q.; Li, J.; Liu, H.; Zhang, Z.; Liu, T.; Liu, Z. Covalent Organic Polymer as a Carborane Carrier for Imaging-Facilitated Boron Neutron Capture Therapy. *ACS Appl Mater Interfaces* **2020**, *12*, 55564–55573, doi:10.1021/acsami.0c15251.
52. Varadarajan, A.; Sharkey, R.M.; Goldenberg, D.M.; Frederick Hawthorne, M. Conjugation of Phenyl Isothiocyanate Derivatives of Carborane to Antitumor Antibody and in Vivo Localization of Conjugates in Nude Mice. *Bioconj Chem*, **1991**, *2*, 102-110, https://doi.org/10.1021/bc00008a005.
53. Paxton, R.J.; Beatty, B.G.; Varadarajan, A.; Hawthorne, M.F. Carboranyl Peptide-Antibody Conjugates for Neutron-Capture Therapy: Preparation, Characterization, and in Vivo Evaluation. *Bioconjug Chem* **1992**, *3*, 241–247, doi:10.1021/bc00015a007.
54. Chen, C.J.; Primus, F.J.; Szalai, G.; Shively, J.E.; Kane, R.R.; Hawthorne, M.F. Synthesis and Characterization of Oligomeric Nido-Carboranyl Phosphate Diester Conjugates to Antibody and Antibody Fragments for Potential Use in Boron Neutron Capture Therapy of Solid Tumors. *Bioconjug Chem* **1994**, *5*, 557–564, doi:10.1021/bc00030a011.
55. Wilbur, D.S.; Chyan, M.K.; Hamlin, D.K.; Vessella, R.L.; Wedge, T.J.; Hawthorne, M.F. Reagents for Astatination of Biomolecules. 2. Conjugation of Anionic Boron Cage Pendant Groups to a Protein Provides a Method for Direct Labeling That Is Stable to in Vivo Deastatination. *Bioconjug Chem* **2007**, *18*, 1226–1240, doi:10.1021/bc060345s.
56. El-Zaria, M.E.; Genady, A.R.; Janzen, N.; Petlura, C.I.; Beckford Vera, D.R.; Valliant, J.F. Preparation and Evaluation of Carborane-Derived Inhibitors of Prostate Specific Membrane Antigen (PSMA). *Dalton Transactions* **2014**, *43*, 4950–4961, doi:10.1039/c3dt53189a.

57. Miyajima, Y.; Nakamura, H.; Kuwata, Y.; Lee, J.D.; Masunaga, S.; Ono, K.; Maruyama, K. Transferrin-Loaded Nido-Carborane Liposomes: Tumor-Targeting Boron Delivery System for Neutron Capture Therapy. *Bioconjug Chem* **2006**, *17*, 1314–1320, doi:10.1021/bc060064k.
58. Genady, A.R.; Tan, J.; El-Zaria, M.E.; Zlitni, A.; Janzen, N.; Valliant, J.F. Synthesis, Characterization and Radiolabeling of Carborane-Functionalized Tetrazines for Use in Inverse Electron Demand Diels-Alder Ligation Reactions. *J Organomet Chem* **2015**, *791*, 204–213, doi:10.1016/j.jorganchem.2015.05.033.
59. Sumitani, S.; Oishi, M.; Nagasaki, Y. Carborane Confined Nanoparticles for Boron Neutron Capture Therapy: Improved Stability, Blood Circulation Time and Tumor Accumulation. *React Funct Polym* **2011**, *71*, 684–693, doi:10.1016/j.reactfunctpolym.2011.03.010.
60. Sumitani, S.; Oishi, M.; Yaguchi, T.; Murotani, H.; Horiguchi, Y.; Suzuki, M.; Ono, K.; Yanagie, H.; Nagasaki, Y. Pharmacokinetics of Core-Polymerized, Boron-Conjugated Micelles Designed for Boron Neutron Capture Therapy for Cancer. *Biomaterials* **2012**, *33*, 3568–3577, doi:10.1016/j.biomaterials.2012.01.039.
61. Gona, K.B.; Zaulet, A.; Gómez-Vallejo, V.; Teixidor, F.; Llop, J.; Viñas, C. COSAN as a Molecular Imaging Platform: Synthesis and "in Vivo" Imaging. *Chemical Communications* **2014**, *50*, 11415–11417, doi:10.1039/c4cc05058d.
62. Kimura, S.; Masunaga, S.I.; Harada, T.; Kawamura, Y.; Ueda, S.; Okuda, K.; Nagasawa, H. Synthesis and Evaluation of Cyclic RGD-Boron Cluster Conjugates to Develop Tumor-Selective Boron Carriers for Boron Neutron Capture Therapy. *Bioorg Med Chem* **2011**, *19*, 1721–1728, doi:10.1016/j.bmc.2011.01.020.
63. Wang, S.; Blaha, C.; Santos, R.; Huynh, T.; Hayes, T.R.; Beckford-Vera, D.R.; Blecha, J.E.; Hong, A.S.; Fogarty, M.; Hope, T.A.; et al. Synthesis and Initial Biological Evaluation of Boron-Containing Prostate-Specific Membrane Antigen Ligands for Treatment of Prostate Cancer Using Boron Neutron Capture Therapy. *Mol Pharm* **2019**, *16*, 3831–3841, doi:10.1021/acs.molpharmaceut.9b00464.
64. Quan, H.; Fan, L.; Huang, Y.; Xia, X.; He, Y.; Liu, S.; Yu, J. Hyaluronic Acid-Decorated Carborane-TAT Conjugation Nanomicelles: A Potential Boron Agent with Enhanced Selectivity of Tumor Cellular Uptake. *Colloids Surf B Biointerfaces* **2021**, *204*, doi:10.1016/j.colsurfb.2021.111826.
65. Feakes, D.A.; Shelly, K.; Hawthorne, M.F. Selective Boron Delivery to Murine Tumors by Lipophilic Species Incorporated in the Membranes of Unilamellar Liposomes. *Proc Natl Acad Sci U S A* **1995**, *92*, 1367–1371, doi:10.1073/pnas.92.5.1367.
66. Heber, E.M.; Kueffer, P.J.; Lee, M.W.; Hawthorne, M.F.; Garabalino, M.A.; Molinari, A.J.; Nigg, D.W.; Bauer, W.; Hughes, A.M.; Pozzi, E.C.C.; et al. Boron Delivery with Liposomes for Boron Neutron Capture Therapy (BNCT): Biodistribution Studies in an Experimental Model of Oral Cancer Demonstrating Therapeutic Potential. *Radiat Environ Biophys* **2012**, *51*, 195–204, doi:10.1007/s00411-011-0399-0.
67. Heber, E.M.; Hawthorne, M.F.; Kueffer, P.J.; Garabalino, M.A.; Thorp, S.I.; Pozzi, E.C.C.; Hughes, A.M.; Maitz, C.A.; Jalisatgi, S.S.; Nigg, D.W.; et al. Therapeutic Efficacy of Boron Neutron Capture Therapy Mediated by Boron-Rich Liposomes for Oral Cancer in the Hamster Cheek Pouch Model. *Proc Natl Acad Sci U S A* **2014**, *111*, 16077–16081, doi:10.1073/pnas.1410865111.
68. Li, T.; Hamdi, J.; Hawthorne, M.F. Unilamellar Liposomes with Enhanced Boron Content. *Bioconjug Chem* **2006**, *17*, 15–20, doi:10.1021/bc0501350.
69. Takeuchi, I.; Tomoda, K.; Matsumoto, K.; Uchiro, H.; Makino, K. PEGylated Liposomes Prepared with Polyborane Instead of Cholesterol for BNCT: Characteristics and Biodistribution Evaluation. *Colloid Polym Sci* **2016**, *294*, 1679–1685, doi:10.1007/s00396-016-3925-4.
70. Kueffer, P.J.; Maitz, C.A.; Khan, A.A.; Schuster, S.A.; Shlyakhtina, N.I.; Jalisatgi, S.S.; Brockman, J.D.; Nigg, D.W.; Hawthorne, M.F. Boron Neutron Capture Therapy Demonstrated in Mice Bearing EMT6 Tumors Following Selective Delivery of Boron by Rationally Designed Liposomes. *Proc Natl Acad Sci U S A* **2013**, *110*, 6512–6517, doi:10.1073/pnas.1303437110.
71. Takeuchi, I.; Kishi, N.; Shiokawa, K.; Uchiro, H.; Makino, K. Polyborane Encapsulated Liposomes Prepared Using PH Gradient and Reverse-Phase Evaporation for Boron Neutron Capture Therapy: Biodistribution in Tumor-Bearing Mice. *Colloid Polym Sci* **2018**, *296*, 1137–1144, doi:10.1007/s00396-018-4331-x.
72. Tsygankova, A.R.; Gruzdev, D.A.; Kanygin, V. V.; Ya. Guselnikova, T.; Telegina, A.A.; Kasatova, A.I.; Kichigin, A.I.; Levit, G.L.; Mechetina, L. V.; Mukhamadiyarov, R.A.; et al. Liposomes Loaded with Lipophilic Derivative of Closo-Carborane as a Potential Boron Delivery System for Boron Neutron Capture Therapy of Tumors. *Mendeleev Communications* **2021**, *31*, 659–661, doi:10.1016/j.mencom.2021.09.022.

73. Xiong, H.; Zhou, D.; Qi, Y.; Zhang, Z.; Xie, Z.; Chen, X.; Jing, X.; Meng, F.; Huang, Y. Doxorubicin-Loaded Carborane-Conjugated Polymeric Nanoparticles as Delivery System for Combination Cancer Therapy. *Biomacromolecules* **2015**, *16*, 3980–3988, doi:10.1021/acs.biomac.5b01311.
74. Takeuchi, I.; Tomoda, K.; Matsumoto, K. PEGylated Liposomes Prepared with Polyborane Instead of Cholesterol for BNCT : Characteristics and Biodistribution Evaluation. *Colloid Polym Sci* **2016**, 1679–1685, doi:10.1007/s00396-016-3925-4.
75. Lee, W.; Sarkar, S.; Ahn, H.; Kim, J.Y.; Lee, Y.J.; Chang, Y.; Yoo, J. PEGylated Liposome Encapsulating Nido-Carborane Showed Significant Tumor Suppression in Boron Neutron Capture Therapy (BNCT). *Biochem Biophys Res Commun* **2020**, *522*, 669–675, doi:10.1016/j.bbrc.2019.11.144.
76. Chen, G.; Yang, J.; Lu, G.; Liu, P.C.; Chen, Q.; Xie, Z.; Wu, C. One Stone Kills Three Birds: Novel Boron-Containing Vesicles for Potential Bnct, Controlled Drug Release, and Diagnostic Imaging. *Mol Pharm* **2014**, *11*, 3291–3299, doi:10.1021/mp400641u.
77. Wang, D.; Meng, Y.; Wang, X.; Xia, G.; Zhang, Q. The Endocytic Mechanism and Cytotoxicity of Boron-Containing Vesicles. *Chem Pharm Bull* **2020**, 68, 618-627, doi: 10.1248/cpb.c19-00971.
78. Zhang, T.; Li, G.; Li, S.; Wang, Z.; He, D.; Wang, Y.; Zhang, J.; Li, J.; Bai, Z.; Zhang, Q.; et al. Asialoglycoprotein Receptor Targeted Micelles Containing Carborane Clusters for Effective Boron Neutron Capture Therapy of Hepatocellular Carcinoma. *Colloids Surf B Biointerfaces* **2019**, *182*, doi:10.1016/j.colsurfb.2019.110397.
79. Fithroni, A.B.; Kobayashi, K.; Uji, H.; Ishimoto, M.; Akehi, M.; Ohtsuki, T.; Matsuura, E. Novel Self-Forming Nanosized DDS Particles for BNCT: Utilizing A Hydrophobic Boron Cluster and Its Molecular Glue Effect. *Cells* **2022**, *11*, doi:10.3390/cells11203307.
80. Zhu, Y.; Lin, Y.; Zhu, Y.Z.; Lu, J.; Maguire, J.A.; Hosmane, N.S. Boron Drug Delivery via Encapsulated Magnetic Nanocomposites: A New Approach for BNCT in Cancer Treatment. *J Nanomater* **2010**, *2010*, doi:10.1155/2010/409320.
81. Oleshkevich, E.; Morancho, A.; Saha, A.; Galenkamp, K.M.O.; Grayston, A.; Crich, S.G.; Alberti, D.; Protti, N.; Comella, J.X.; Teixidor, F.; et al. Combining Magnetic Nanoparticles and Icosahedral Boron Clusters in Biocompatible Inorganic Nanohybrids for Cancer Therapy. *Nanomedicine* **2019**, *20*, doi:10.1016/j.nano.2019.03.008.
82. Wang, J.; Chen, L.; Ye, J.; Li, Z.; Jiang, H.; Yan, H.; Stogniy, M.Y.; Sivaev, I.B.; Bregadze, V.I.; Wang, X. Carborane Derivative Conjugated with Gold Nanoclusters for Targeted Cancer Cell Imaging. *Biomacromolecules* **2017**, *18*, 1466–1472, doi:10.1021/acs.biomac.6b01845.
83. Takeuchi, I.; Nomura, K.; Makino, K. Hydrophobic Boron Compound-Loaded Poly(L-Lactide-Co-Glycolide) Nanoparticles for Boron Neutron Capture Therapy. *Colloids Surf B Biointerfaces* **2017**, *159*, 360–365, doi:10.1016/j.colsurfb.2017.08.002.
84. Wang, Y.; Xu, Y.; Yang, J.; Qiu, X.; Li, N.; Zhu, Y.; Yan, L.; Li, W.; Huang, X.; Liang, K.; et al. Carborane Based Mesoporous Nanoparticles as a Potential Agent for BNCT. *Mater Chem Front* **2021**, *5*, 2771–2776, doi:10.1039/d0qm00867b.
85. Zhang, T.; Xu, D.; Yi, Y.; Wang, Y.; Cui, Z.; Chen, X.; Ma, Q.; Song, F.; Zhu, B.; Zhao, Z.; et al. Chitosan-Lactobionic Acid-Thioctic Acid-Modified Hollow Mesoporous Silica Composite Loaded with Carborane for Boron Neutron Capture Therapy of Hepatocellular Carcinoma. *Mater Des* **2022**, *223*, 111196, doi:10.1016/j.matdes.2022.111196.
86. Yinghuai, Z.; Peng, A.T.; Carpenter, K.; Maguire, J.A.; Hosmane, N.S.; Takagaki, M. Substituted Carborane-Appended Water-Soluble Single-Wall Carbon Nanotubes: New Approach to Boron Neutron Capture Therapy Drug Delivery. *J Am Chem Soc* **2005**, *127*, 9875–9880, doi:10.1021/ja0517116.
87. Pulagam, K.R.; Gona, K.B.; Gomez-Vallejo, V.; Meijer, J.; Zilberfain, C.; Estrela-lopis, I.; Baz, Z.; Cossio, U.; Llop, J. Gold Nanoparticles as Boron Carriers for Boron and In Vivo Evaluation. *Molecules* **2019**, *24*, 3609.
88. Pulagam, K.R.; Henriksen-Lacey, M.; B. Uribe, K.; Renero-Lecuna, C.; Kumar, J.; Charalampopoulou, A.; Facoetti, A.; Protti, N.; Gómez-Vallejo, V.; Baz, Z.; et al. In Vivo Evaluation of Multifunctional Gold Nanorods for Boron Neutron Capture and Photothermal Therapies. *ACS Appl Mater Interfaces* **2021**, *13*, 49589–49601, doi:10.1021/acsami.0c17575.
89. Ferrer-Ugalde, A.; Sandoval, S.; Pulagam, K.R.; Muñoz-Juan, A.; Laromaine, A.; Llop, J.; Tobias, G.; Núñez, R. Radiolabeled Cobaltabis(Dicarbollide) Anion-Graphene Oxide Nanocomposites for in Vivo Bioimaging and Boron Delivery. *ACS Appl Nano Mater* **2021**, *4*, 1613–1625, doi:10.1021/ACSANM.0C03079/ASSET/IMAGES/LARGE/AN0C03079_0007.JPEG.

90. Shi, Y.; Guo, Z.; Fu, Q.; Shen, X.; Zhang, Z.; Sun, W.; Wang, J.; Sun, J.; Zhang, Z.; Liu, T.; et al. Localized Nuclear Reaction Breaks Boron Drug Capsules Loaded with Immune Adjuvants for Cancer Immunotherapy. *Nat Commun* **2023**, *14*, 1–15, doi:10.1038/s41467-023-37253-x.
91. Setiawan, Y.; Moore, D.E.; Allen, B.J. Selective Uptake of Boronated Low-Density Lipoprotein in Melanoma Xenografts Achieved by Diet Supplementation. *Br J Cancer* **1996**, *74*, 1705–1708, doi:10.1038/bjc.1996.618.
92. Xiong, H.; Wei, X.; Zhou, D.; Qi, Y.; Xie, Z.; Chen, X.; Jing, X.; Huang, Y. Amphiphilic Polycarbonates from Carborane-Installed Cyclic Carbonates as Potential Agents for Boron Neutron Capture Therapy. *Bioconjug Chem* **2016**, *27*, 2214–2223, doi:10.1021/acs.bioconjchem.6b00454.
93. Fuentes, I.; García-Mendiola, T.; Sato, S.; Pita, M.; Nakamura, H.; Lorenzo, E.; Teixidor, F.; Marques, F.; Viñas, C. Metallacarboranes on the Road to Anticancer Therapies: Cellular Uptake, DNA Interaction, and Biological Evaluation of Cobaltabisdicarbollide [COSAN]−. *Chemistry - A European Journal* **2018**, *24*, 17239–17254, doi:10.1002/chem.201803178.
